# The Effect of Maternal Folic Acid Supplementation on Neurodevelopmental Disorders in Offspring: An Umbrella Review of Systematic Reviews and Meta-Analyses

**DOI:** 10.3390/nu17213443

**Published:** 2025-10-31

**Authors:** Miao Yu, Yiming Hu, Lei Hou, Xiaomin Wu, Xiangxin Chen, Ruohan Yan, Jie Dong, Jing Wu

**Affiliations:** 1 School of Public Health, Inner Mongolia Medical University, Hohhot 010110, China; ym15304746119@outlook.com; 2 National Center for Chronic and Noncommunicable Disease Control and Prevention, Chinese Center for Disease Control and Prevention, Beijing 100050, China; yiminghu_hym@outlook.com (Y.H.); houlei@ncncd.chinacdc.cn (L.H.); wxm250258@163.com (X.W.); 13296932016@163.com (X.C.); yanrh1207@163.com (R.Y.); dongjie137741@163.com (J.D.); 3 School of Public Health, Baotou Medical College, Inner Mongolia University of Science & Technology, Baotou 014040, China; 4 School of Public Health, Tianjin Medical University, Tianjin 300070, China; 5 School of Public Health, China Medical University, Shenyang 110000, China

**Keywords:** folic acid, neurodevelopmental disorders, offspring, umbrella review

## Abstract

**Objectives**: Maternal folic acid supplementation is recommended to prevent neural tube defects (NTDs), yet its influence on offspring neurodevelopmental disorders (NDDs) remains uncertain. This umbrella review aims to evaluate whether maternal folic acid supplementation before and/or during pregnancy affects the risk of NDDs. **Methods:** We conducted a systematic search in MEDLINE, Cochrane Library, EMBASE, PubMed, and Web of Science from inception to 30 June 2025, to identify systematic reviews (SRs) and meta-analyses (MAs) that synthesized evidence from non-randomized studies on maternal folic acid supplementation and NDDs. Methodological quality was assessed using the AMSTAR-2 assessment and evidence certainty using the GRADE framework. **Results:** A total of 23 SRs/MAs were included, of which 14 did not perform meta-analysis. Most included SRs/MAs were methodologically limited, with 50.00% rated as very low quality and only 36.37% achieving high or moderate quality. MAs indicated a protective effect of supplementation, with odds ratio (OR) of 0.66 (95% confidence interval (CI): 0.55–0.79) for autism spectrum disorder (ASD), 0.86 (95% CI: 0.78–0.95) for attention-deficit/hyperactivity disorder (ADHD), and 0.75 (95% CI: 0.63–0.91) for behavioral problems. No significant associations were found for motor, intellectual/cognitive, or language development. SRs reported inconsistent conclusions across most outcomes. **Conclusions:** In summary, maternal folic acid supplementation may reduce the risk of ASD, ADHD, and behavioral problems in offspring. Although the current evidence is of low quality, supplementation guidelines are justified by the well-established benefits for NTDs. Further research is required to address remaining uncertainties.

## 1. Introduction

Neurodevelopmental disorders (NDDs) are a group of patients with behavioral and cognitive disorders that occur during the developmental period. These disorders primarily affect the acquisition and progression of intellectual, motor, linguistic, and social abilities [1]. As defined by the *Diagnostic and Statistical Manual of Mental Disorders, Fifth Edition* (DSM-5) and the 10th/11th revisions of the International Classification of Diseases (ICD-10/11), common NDDs include Autism Spectrum Disorder (ASD), Attention-Deficit/Hyperactivity Disorder (ADHD), Intellectual Developmental Disorder (IDD), Tic Disorders (TD), Developmental Speech or Language Disorders, Specific Learning Disorder, and related conditions [2]. The prevalence of NDDs in the general population is approximately 5% to 7%. Onset typically occurs in early childhood, and symptoms can persist throughout the lifespan, frequently resulting in significantly higher rates of unemployment among affected individuals [3]. Due to their early onset and the frequent need for long-term support that may extend throughout life, NDDs pose considerable social, economic, and healthcare challenges.

Folic acid, a crucial cofactor in one-carbon metabolism, plays an essential role in amino acid metabolism, nucleotide synthesis, and methylation processes [4]. Folic acid supplementation during pregnancy has been proven to effectively reduce the risk of neural tube defects (NTDs) in offspring [5,6,7,8]. To further reduce the global incidence of NTDs, the World Health Organization issued guidelines in 2015 recommending that countries implement public health measures to ensure adequate folic acid levels among women of reproductive age [9]. Although the protective effect of folic acid against NTDs is well established, its potential influence on a broader range of NDDs remains controversial. Some systematic reviews (SRs) and meta-analyses (MAs) indicate that maternal folic acid supplementation could potentially reduce the risk of ASD, ADHD, or other neurodevelopmental conditions [10,11,12,13]. In contrast, other studies have reported no significant protective association or have concluded that the evidence remains inconclusive [12,14,15,16].

In summary, current SRs and MAs present inconsistent conclusions on this topic. Due to the early onset and chronic course of neurodevelopmental disorders, early intervention—including potential intrauterine strategies—may be critically important. This review synthesizes evidence from existing SRs and MAs to further elucidate the relationship between maternal folic acid supplementation and the risk of neurodevelopmental disorders in offspring, thereby informing early intervention strategies for these conditions.

## 2. Materials and Methods

### 2.1. Registration and Protocol

This umbrella review was conducted in accordance with the PRISMA 2020 guidelines [17] and has been registered with the International Prospective Register of Systematic Reviews (PROSPERO), registration number: CRD420251020920.

### 2.2. Eligibility Criteria for the Studies

#### 2.2.1. Outcomes

Neurodevelopmental disorders in offspring, including ASD, ADHD, IDD, TD, and other neurodevelopmental conditions.

#### 2.2.2. Inclusion Criteria

SRs and MAs were included if they met the following criteria: (1) they were SRs or MAs of non-randomized studies, such as observational or non-randomized interventional studies; (2) they evaluated the association between maternal folic acid supplementation before and/or during pregnancy and any neurodevelopmental outcomes in offspring; (3) and they were published in English or Chinese.

#### 2.2.3. Exclusion Criteria

We will exclude animal studies, laboratory studies, and SRs and/or MAs evaluating the effect of polymorphisms in genes involved in folate metabolism on the outcome of offspring neurodevelopmental disorders.

### 2.3. Information Sources and Search Strategies

On 30 June 2025, a comprehensive literature search was conducted following the PICO framework. Controlled vocabulary and free-text terms were used to systematically search for relevant SRs and/or MAs in MEDLINE, the Cochrane Library, EMBASE (Ovid), PubMed, and Web of Science from inception. The specific search strategies are detailed in Supplementary Materials. Two reviewers independently screened the titles, abstracts, and full texts of potentially eligible studies. Any disagreements were resolved by a third reviewer.

### 2.4. Studies Selection and Extraction of Data

If an SR and/or MA addresses multiple neurodevelopmental disorders, each relevant analysis was included separately. If an SR and/or MA incorporated only a single original study pertinent to the research topic, it was still included in the characteristics table; however, it was verified whether that original study had been redundantly included in other SRs and/or MAs. In cases of duplication, the SR and/or MA with superior methodological quality or broader scope was selected as the representative source. SR and/or MA that were superseded in this manner would be retained solely in the characteristics table to reflect methodological heterogeneity and did not contribute to subsequent qualitative or quantitative evidence synthesis.

For the included SRs and MAs, two researchers independently extracted data—including the first author’s name, year of publication, folic acid dosage and duration, and key outcomes such as effect sizes or primary conclusions—using a standardized data extraction form. Any discrepancies between extractors were resolved through discussion or by arbitration from a third researcher.

### 2.5. Quality Assessment

To ensure methodological rigor, two researchers independently evaluated the methodological quality of the included SRs and MAs using the latest version of AMSTAR-2 (A Measurement Tool to Assess SRs) [18]. This tool consists of 16 domains that assess key aspects of review methodology. Any disagreements between assessors were resolved through discussion or by a third researcher to ensure consistency and objectivity. The overall confidence ratings for each review were categorized into one of four levels: high, moderate, low, or critically low (CL). The AMSTAR-2 assessment results are provided in Supplementary Materials, Table S6.

### 2.6. Credibility of the Evidence

The GRADE (Grading of Recommendations, Assessment, Development, and Evaluations) framework was employed to evaluate the quality of evidence from SRs and MAs included in umbrella reviews [19,20]. GRADE classifies the certainty of evidence into four levels: high, moderate, low, and very low. Within this system, evidence from randomized controlled trials (RCTs) is initially rated as high quality, whereas evidence from observational studies is initially rated as low quality. The rating may then be upgraded or downgraded based on specific criteria. For example, unexplained heterogeneity or publication bias may lower the certainty of evidence, while a large magnitude of effect or a dose–response relationship may increase it. Two independent reviewers performed the assessments, with any disagreements resolved through discussion or by arbitration from a third researcher.

### 2.7. Statistical Analysis

The overall effect sizes and 95% (CI) from each MA were reanalyzed using the DerSimonian and Laird (DL) random-effects model. We calculated the Higgins and Thompson *I*-square (*I*^2^) statistic, Cochran’s Q test *p*-value for heterogeneity, and Egger’s regression test *p*-value for publication bias. Heterogeneity was assessed using the *I*^2^ statistic, with values of 25%, 50%, and 75% representing low, moderate, and high heterogeneity, respectively [21]. An excess significance test was performed to evaluate whether the number of statistically significant studies (O) exceeds the expected number (E). Small-study effects were examined using Egger’s regression asymmetry test, with statistical significance defined as *p* < 0.10. If more recent MAs omitted original studies included in previous analyses, the data were pooled and reanalyzed. All statistical analyses were conducted using R software, version 4.3.1.

## 3. Results

The initial literature search was conducted across five major databases (PubMed, Embase, Medline, Cochrane Library, and Web of Science), identifying 429 potentially relevant records. After removing duplicates, 273 unique records were retained for title and abstract screening.

During the initial screening phase, 46 records were excluded according to predefined criteria: 10 were conference abstracts, case reports, guidelines, or correspondence; the remaining 36 were excluded as they were not SRs or MAs. Subsequently, a full-text eligibility assessment was performed on 227 articles. Through manual screening of the reference lists of included studies, 3 additional records were identified. Following full-text assessment, 198 articles were excluded for the following reasons: ineligible intervention population (n = 40), exposure unrelated to folic acid (n = 65), and inconsistent outcome definitions (n = 93). Additionally, 9 records were excluded due to being compilations of conference abstracts (n = 5), inability to isolate folic acid effects from multivitamin supplementation (n = 1), or because the included studies are SRs and RCTs (n = 3).

**Figure 1:**
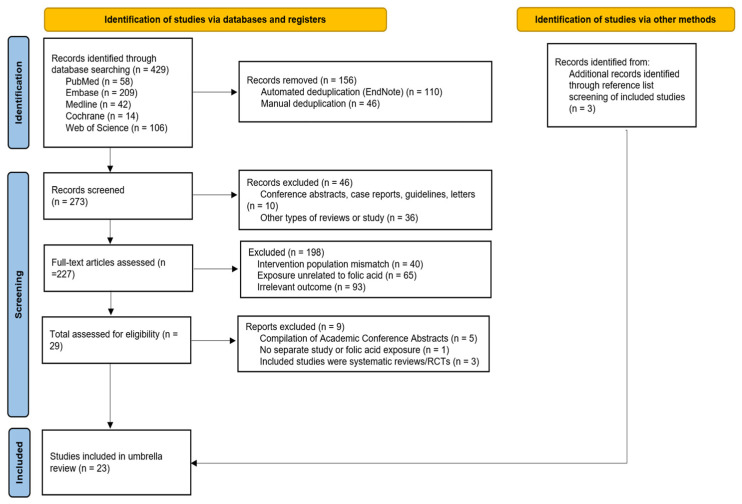
Flow diagram of studies selected for inclusion.

This umbrella review included a total of 23 SRs and MAs (Figure 1). Among these, Vasconcelos et al. [16] constitutes an updated analysis of Castro et al. [22]. Consequently, 22 SRs and/or MAs—encompassing 44 individual studies after accounting for those reporting multiple outcomes separately—were included in the final evaluation and analysis.

### 3.1. AMSTAR-2 Assessment of Included Systematic Reviews

Based on the AMSTAR-2 assessment, 7 SRs and MAs (31.82%) were rated as high quality, 1 (4.55%) as moderate quality, 3 (13.64%) as low quality, and 11 (50.00%) as CL quality. Further details are provided in Supplementary Materials, Table S6.

### 3.2. GRADE Assessment of Interventions

As this umbrella review incorporates SRs and MAs of non-randomized controlled trials, the initial GRADE rating for the overall evidence was low. The majority of studies were at risk of bias, primarily due to dependence on retrospective methods for exposure assessment. Among the 18 SRs and MAs addressing ASD outcomes, only Liu et al. [10] was upgraded to moderate quality, owing to large effect sizes and other reinforcing factors; the remaining 3 were rated as low quality and 14 as very low quality. For intellectual or cognitive development outcomes, only Veena et al. [23] provided moderate-quality evidence, while all others were of very low quality. Similarly, for language development, only Veena et al. [23] offered moderate-quality evidence. Evidence pertaining to ADHD, behavioral, motor, and mental development, as well as neurodevelopmental outcomes, was consistently rated as low or very low quality.

### 3.3. Meta-Analysis

As indicated in Table 1, meta-analyses were performed in 9 of the included SRs. Among these, 9 articles reported outcomes related to ASD, 2 focused on motor development, and 1 each addressed outcomes pertaining to ADHD, mental development, language development, behavioral development, and intellectual or cognitive development.

To address methodological inconsistencies in the original studies, we identified robust associations by re-evaluating all MAs using the DL random-effects model. Summary effect sizes and 95% confidence intervals (CIs) were recalculated, and heterogeneity was quantified using *I*^2^ statistics and Cochran’s Q test. Egger’s regression test and the excess significance test were applied under the following conditions: (1) a pooled effect *p*-value < 0.05; (2) inclusion of at least 4 studies for the excess significance test (otherwise denoted as “NA”) [24]; and (3) inclusion of at least 3 studies for Egger’s test (otherwise “NA”) [25]. Detailed results are provided in Supplementary Materials, Table S8. Among the 14 recalculated MAs, 9 (64.29%) showed significant heterogeneity (*I*^2^ > 50% and Q test *p* < 0.05). The highest heterogeneity was observed for ASD, with *I*^2^ values ranging from 60.2% to 97.3%. Of the 8 analyses eligible for publication bias assessment, 3 yielded significant Egger’s test results (*p* < 0.10), suggesting the potential publication bias. Furthermore, excess significance testing indicated that 6 analyses had an over-representation of statistically significant positive results.

When multiple SRs or MAs addressing similar research questions include the same original studies, a phenomenon known as literature overlap occurs. Directly synthesizing such overlapping studies may disproportionately weight those original studies that are included more frequently, potentially introducing bias and undermining the accuracy and reliability of the conclusions [26]. Therefore, in this umbrella review, the Corrected Covered Area (*CCA*) [27] method is applied to quantitatively assess the degree of overlap among the included MAs.

CCA=(N−r)/(r×c−r)

A *CCA* value of ≤5% is considered to indicate slight overlap; 6–10% represents moderate overlap; 11–15% denotes a high degree of overlap; and ≥15% suggests a very high degree of overlap. When the *CCA* reaches or exceeds 6%, the following strategies are implemented to manage study overlap [28]: If multiple MAs were published more than 24 months apart, the most recent one is selected for data extraction and analysis. For those published within a 24-month window, the MA with the highest AMSTAR-2 score is prioritized. If scores are identical, the one providing the most comprehensive information is selected for further analysis.

**Table 1:** Characteristics of included meta-analyses.

Author (year)	Exposure		Exposure Comparison	Assessment Criteria/Tool	Study Design (Number of Studies)	No. of Participants (Cases)	Databases Searched	Locations	Statistical Model/Effect Size Indicator (95% CI)
	Dose	Window							
ASD									
Wang et al. [11]	NA	Before and/or during pregnancy	No folic acid supplementation	DSM-IV, CARS, ICD-10, ADI-R, ADOS, etc.	All: 12 Cohort studies: 4 RR = 0.903 (0.786–0.998) Case–control studies: 6 RR = 0.435 (0.264–0.717)	631,905 (4514)	PubMed, Web of Knowledge, and Wanfang Database (as recent as March 2017)	Asian, European, American	Random effects model/RR = 0.771 (0.641–0.928)
Guo et al. [14]	NA	Periconception period, early pregnancy, mid-pregnancy, pre-pregnancy	No folic acid supplementation	DSM-IV, DSM-IV-TR, ICD-9/10, ADI-R, ADOS, ADOS-G	All: 8 Prospective cohort studies: 4 Registry-based cohort study: 1 Case–cohort study: 1 Case–control studies: 2	840,776 (7127)	PubMed, EMBASE, PsycINFO, Scopus, Web of Science, and Cochrane Library (up to 7 June 2018)	Denmark, Norway, Sweden, the United States, and Israel	Random effects model/OR = 0.91 (0.73–1.13) and HR = 0.66 (0.38–1.17)
Yu et al. [29]	NA	Before pregnancy and early pregnancy	No folic acid supplementation	Diagnostic reference standards with internationally recognized or expert consensus are available	All: 10 Cohort studies: 7 Case–control studies: 3	1,230,294 (4459)	PubMed, Embase, Scopus, Cochrane Library, EBSCO, CNKI, Wanfang Data, VIP, and CBD	China, Norway, Denmark, the United States of America, The Netherlands, Oman	Random effects model/OR = 0.798 (0.669–0.952)
Liu et al. [10]	No detail doses; detail doses: ≥400 µg/d, ≥500 µg/d, ≥800 µg/d	Before pregnancy (12 weeks before the start of pregnancy); early pregnancy (start of pregnancy-12 weeks after); during pregnancy (no specific pregnancy and folic acid exposure period occurring at the period from 270 days before childbirth up to the date of delivery); before pregnancy to early pregnancy	No folic acid supplementation	DSM-IV, ICD-8/9/10, ADOS, ADI-R, SCQ	All: 10 Cohort studies: 6 Case–control studies: 4	NA (9795)	PubMed, EMBASE, and Cochrane Library (Until 31 January 2020)	European countries, the United States of America, Israel, and China	Random effects model/OR _during the prenatal period_ = 0.570 (0.460–0.720)
Jia et al. [30]	NA	Pre-pregnancy and pregnancy, early pregnancy, 4 weeks before pregnancy to 8 weeks after pregnancy	No folic acid supplementation	DSM-V/IV, ICD-10, CARS	All: 17 Cohort studies: 8 Case–control studies: 9	887,053 (10,812)	PubMed, Embase, Web of Science, The Cochrane Library, Scopus, CNKI, WanFang Data, VIP, and CBM (31 December 2020)	China, Israel, Norway, Sweden, the Netherlands, Oman, the United States, Denmark	Random effects model/OR = 0.890 (0.770–1.030)
Vazque et al. [15]	NA	Periconception and early pregnancy	No folic acid supplementation	ADI-R, ADOS, SRS, DSM-V/IV, ICD-9/10	About: 9 Cohort studies: 6 Case–control studies: 3	739,226 (6396)	PubMed, Scopus, and The Cochrane Library (until June 2018)	The United States, Norway, Denmark, Sweden, and Israel	Random effects model/OR = 0.580 (0.460–0.750)
Friel et al. [31]	NA	Periconception period, early pregnancy, and pregnancy	Low or no supplement intake	ICD-9/10, DSM, etc.	About: 10 Cohort studies: 4 Case–control studies: 3 Nested case–control studies: 2 Population-based cohort and sibling case–control: 1	904,947 (8159)	MEDLINE (OVID), EMBASE (OVID), PsycINFO (EBSCO), Web of Science core collection, Open gray, and BioRix (until 8 June 2020)	Sweden, the United States, Israel, China, Denmark, Norway	Random effects model/RR = 0.740 (0.530–1.040)
Li et al. [32]	NA	Periconception period, early pregnancy, and pregnancy	No folic acid supplementation and/or normal diet	Population-based patient registries, medical records, ASD diagnosis or screening, and service registries	About: 6 Cohort studies: 6	528,810 (NA)	PubMed and Embase (through March 2019)	The United States, Israel, Denmark, Sweden, and Norway	Random effects model/RR = 0.640 (0.460–0.900)
Chen et al. [12]	NA	Before pregnancy, early pregnancy, pregnancy	No use, the lowest dose, below the recommended dose, or the shortest period	CABS, DSM-IV, ICD-10/9, ADI-R, ADOS, ABC, etc.	About: 16 Cohort studies: 8 Case–control studies: 8	360,282 (NA)	MEDLINE, Web of Science, Cochrane Library, Scopus, EMBASE, and PsychInfo (until 24 September 2021)	China, Sweden, Israel, Norway, the United States, Denmark, Saudi Arabia	Case–control studies: random effects model/OR = 0.670 (0.430–1.050) Cohort studies: random effects model/OR = 0.690 (0.510–0.930)
ADHD									
Chen et al. [12]	NA	Before and/or during pregnancy	No use, the lowest dose, below the recommended dose, or the shortest period	SDQ Preschool Version, CPT-II Omission errors, DSM-IV, etc.	About: 6 Cohort studies: 6	35,402 (NA)	MEDLINE, Web of Science, Cochrane Library, Scopus, EMBASE, and PsychInfo (until 24 September 2021)	New Zealand, Spain, Japan, Denmark	Fixed effect model/OR = 0.860 (0.780–0.950)
Motor Development									
Chen et al. [12]	NA	Before and/or during pregnancy	No use, the lowest dose, below the recommended dose, or the shortest period	BSID-II, MCSA, Bayley test, the psychomotor scale (Ps) of BSID-I	About: 4 Cohort studies: 8	3424 (NA)	MEDLINE, Web of Science, Cochrane Library, Scopus, EMBASE, and PsychInfo (until 24 September 2021)	Spain, Poland, Mexico	Random effects model/Beta = 1.02 (−0.890–2.920)
Vazque et al. [15]	400–1000 μg/daily, 1000–5000 μg/daily, 5000 μg/daily	Periconception and early pregnancy	Less than 400 μg/d	Bayley, McCarthy	About: 4 Cohort studies: 4	8804 (NA)	PubMed, Scopus, and The Cochrane Library (until June 2018)	Greece, Spain, Poland	Random effects model/SMD = −0.020 (−0.080–0.040)
Intellectual or cognitive development									
Chen et al. [12]	NA	Before and/or during pregnancy	No use, the lowest dose, below the recommended dose, or shortest period	Bayley-III, MCSA, BSID, etc.	About: 5 Cohort studies: 5	4910 (NA)	MEDLINE, Web of Science, Cochrane Library, Scopus, EMBASE, and PsychInfo (until 24 September 2021)	Greece, Spain, Poland, China	Random effects model/Beta = 1.30 (−1.610–4.210)
Behavior development									
Chen et al. [12]	NA	Before and/or during pregnancy	No use	SDQ, CBCL, etc.	About: 3 Cohort studies: 3	36,275 (NA)	MEDLINE, Web of Science, Cochrane Library, Scopus, EMBASE and PsychInfo (until 24 September 2021)	New Zealand, The Netherlands, Denmark	Fixed effect model/OR = 0.750 (0.630–0.910)
Language development									
Chen et al. [12]	NA	Before and/or during pregnancy	No use, the lowest dose, below the recommended dose, or the shortest period	Bayley test, CDIs, MCSA, etc.	About: 4 Cohort studies: 4	8408 (NA)	MEDLINE, Web of Science, Cochrane Library, Scopus, EMBASE, and PsychInfo (until 24 September 2021)	New Zealand, Poland, Spain	Random effects model/Beta = 0.780 (−1.170–2.720)
Mental Development									
Vazque et al. [15]	400–1000 μg/d, 1000–5000 μg/d, 5000 μg/d	Periconception, early pregnancy, and pregnancy	Less than 400 μg/d	Bayley, Popper–Szondi functional development test, Brunet–Lézine Scales, etc.	About: 7 RCT: 2 Cohort studies: 5	11,302 (NA)	PubMed, Scopus, and The Cochrane Library (until June 2018)	Hungary, Germany, Spain, Greece, Poland	Random effects model/SMD = −0.060 (−0.110–0.000)

NA = Not Available; About = Only the original studies relevant to the topic that were included in meta-analyses have been extracted; DSM-V/IV = Diagnostic and Statistical Manual of Mental Disorders (Fifth/Fourth Edition); DSM-IV-TR = Diagnostic and Statistical Manual of Mental Disorders, Fourth Edition, Text Revision; CARS = Childhood Autism Rating Scale; ICD-8/9/10 = International Classification of Diseases-8/9/10; ABC = Autism Behavior Checklist; ADI-R = Autism Diagnostic Interview-Revised; ADOS (-G) = Autism Diagnostic Observation Schedule (-Generic); SCQ = Social Communication Questionnaire; SRS = Social Responsiveness Scale; CABS = Clancy Autism Behavior Scale; SDQ Preschool Version = Strengths and Difficulties Questionnaire for preschool children; CPT-II Omission errors = Conners’ Continuous Performance Test-II Omission errors; BSID-II = Bayley Scales of Infant Development-Second Edition; MCSA = Motor Current Signature Analysis; the psychomotor scale (Ps) of BSID-I = Psychomotor Developmental Index of Bayley Scales of Infant Development-I; McCarthy = McCarthy Scales of Children’s Abilities; SDQ = Strengths and Difficulties Questionnaire; CBCL = Child Behavior Check List; CDIs = Communicative Development Inventories; EBSCO = Elton B. Stephens Company; CNKI = China National Knowledge Infrastructure; VIP = Veipu; CBD = Chinese Biological Databases; CBM = China Biomedical Literature Database; HR = Hazard Ratio; RR = Risk Ratio; OR = Odds Ratio; SMD = Standardized Mean Difference.

#### 3.3.1. ASD

**Figure 2:**
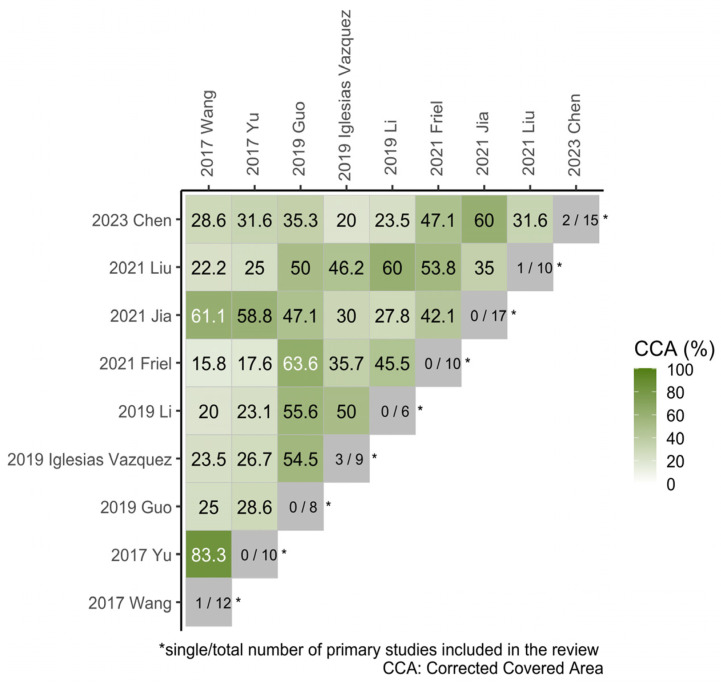
Heatmap of overlapping visualizations of original studies per pair of MAs (ASD). (*CCA* = 0% indicates no overlap of original studies [white] and *CCA* = 100% indicates complete overlap of original studies between meta [dark green]. The gray diagonal tiles present the single/total number of primary studies that included in each review.) [10,11,12,14,15,29,30,31,32].

A review of 9 MAs on ASD outcomes revealed that 5 reported a negative association between maternal folic acid supplementation during pregnancy and offspring ASD incidence, while the remaining 4 showed no significant association. The original studies included in the MAs on ASD exhibited a high degree of overlap (*CCA* = 34.10%), which precluded direct data pooling. Figure 2 illustrates the extent of overlap in original studies between each pair of MAs. Based on the predetermined selection strategy, the MA by Chen et al. [12] was selected for data extraction. Additional original studies not included in that MA were also incorporated. The final data extraction results are presented in Supplementary Materials, Table S9. The study by Li et al. [33] from Chen et al. [12] was excluded due to its animal experimental design. Similarly, the studies by Steenweg-de Graaff et al. [34] and Al-Farsi et al. [35] from Yu et al. [29] were excluded because their exposure factors did not involve maternal folic acid supplementation.

Among the included studies, several—such as Schmidt et al. (2017) [36]—reported multiple effect sizes according to variations in folic acid dosage, timing of supplementation, and duration of follow-up for child outcomes. To avoid violating the assumption of independence and ensure that each study contributed only one effect size to the MA, we selected the most clinically relevant estimate based on the following pre-specified criteria:

1. Folic acid supplementation dosage: preference was given to groups receiving doses closest to the recommended 400 μg/d for neural tube defect prevention [37];
2. Timing of exposure: data reflecting prenatal exposure were prioritized, as this period is most critical for neurodevelopment related to ASD [38];
3. Follow-up duration: outcomes with the longest follow-up (e.g., 5 years) were selected to enhance diagnostic stability.

For studies reporting effect estimates across multiple ASD subtypes, such as Surén et al. [39], the autism disorder (AD) subtype was included in the primary analysis due to its higher clinical representativeness. Effect sizes corresponding to other subtypes (e.g., Asperger syndrome (AS) and pervasive developmental disorder not otherwise specified (PDD-NOS)) were analyzed separately in sensitivity analyses.

**Figure 3:**
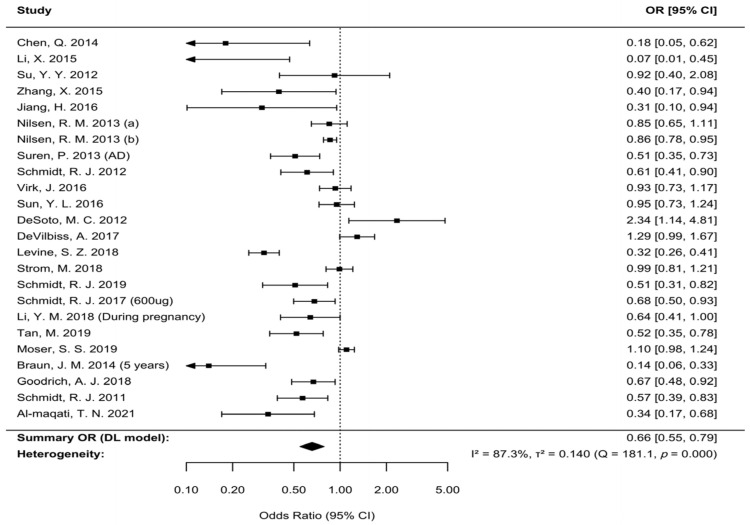
Forest plot of the association between maternal folic acid supplementation and offspring ASD. Individual study estimates are denoted by squares, with horizontal lines indicating 95% CIs; arrows represent truncated CIs. The diamond represents the pooled effect, with its center and width corresponding to the point estimate and 95% CI, respectively. Statistical significance is determined by the position of the diamond relative to the null line (OR = 1). Estimates (a) and (b) from Nilsen et al. (2013) correspond to case–control and cohort study, respectively [11,36,39,40,41,42,43,44,45,46,47,48,49,50,51,52,53,54,55,56,57,58,59].

We used the DL random-effects model, implemented with the R “metafor” package, to synthesize data from 24 studies. The MA indicated that maternal folic acid supplementation is associated with a significant reduction in the risk of offspring ASD (pooled OR = 0.66, 95% CI: 0.55–0.79), as shown in the forest plot (Figure 3). However, significant heterogeneity was detected among the included studies (*I*^2^ = 87.30%, Q = 181.09, *p* = 1.59 × 10^−26^). Furthermore, Egger’s regression test suggested the presence of publication bias (*p* = 0.01). Excess significance testing also indicated an overrepresentation of positive results (O/E = 4.40, *p* = 7.63 × 10^−16^).

##### Subgroup Analyses

Subgroup analyses (Supplementary Materials, Table S10) revealed a comparable protective effect in both case–control studies (13 studies; OR = 0.69, 95% CI: 0.54–0.88; *I*^2^ = 80.2%) and cohort studies (9 studies; OR = 0.65, 95% CI: 0.48–0.88; *I*^2^ = 93.3%). A stronger protective association was observed in cross-sectional studies (OR = 0.24, 95% CI: 0.11–0.56; *I*^2^ = 0.0%); however, this finding should be interpreted cautiously owing to the limited number of included studies (n = 2).

Sample size stratification: Based on tertiles of sample sizes from the 24 included studies, the studies were categorized into three groups: Small (≤651), Medium (652–10,678), and Large (>10,678). The results showed that, compared to the large group, the small group demonstrated a stronger protective effect and lower heterogeneity (OR = 0.47, 95% CI: 0.34–0.65, *I*^2^ = 63.5%), suggesting that sample size may be a source of heterogeneity.

Dose subgroup: No statistically significant differences in effect sizes between the NA group (19 studies; OR = 0.63, 95% CI: 0.50–0.79; *I*^2^ = 87.7%) and the >400 μg/d group (5 studies; OR = 0.71, 95% CI: 0.51–0.99; *I*^2^ = 84.6%). However, due to the substantial heterogeneity observed within both subgroups and the limited number of studies in the >400 μg/d category, these results should be interpreted with caution.

Supplementary period grouping: Although the between-group differences in effect sizes across supplementation periods were not statistically significant, a clear gradient in heterogeneity was observed: periconceptional < early pregnancy < pregnancy. This pattern suggests that later supplementation timing may contribute to greater heterogeneity, though further studies are needed to confirm this association.

Geographical region: This subgroup exhibited varying degrees of regional differences and heterogeneity characteristics. With the exception of the Nordic region (6 studies; OR = 0.90, 95% CI: 0.76–1.06, *I*^2^ = 72.2%), which showed no significant protective effect, all other regions demonstrated beneficial associations. The summary estimate for the Middle East (2 studies; OR = 0.32, 95% CI: 0.26–0.40, *I*^2^ = 0.0%) differed significantly from those of East Asia, Scandinavia, and North America. However, due to the limited number of studies from the Middle East, this finding should be interpreted with caution.

Subgroup analysis based on mandatory folic acid supplementation policies showed no statistically significant difference in effect sizes between the implementation group (8 studies; *I*^2^ = 87.8%) and the non-implementation group (16 studies; *I*^2^ = 87.4%; *p* = 0.8312). When stratified by methodological quality, the pooled effect sizes from high-quality MAs (19 studies; *I*^2^ = 88.5%) would not differ significantly from those derived from low-quality MAs (5 studies; *I*^2^ = 70.0%; *p* = 0.1615). Notably, all subgroups exhibited substantial heterogeneity (*I*^2^ ≥ 70.0%), irrespective of policy status or methodological quality.

##### Sensitivity Analysis

Substituting dosage, exposure time window, or follow-up duration in pediatric populations (e.g., Schmidt et al. [36]: 800 μg/d group, OR = 0.66, 95% CI: 0.55–0.79, *I*^2^ = 87.30%, *p* < 0.001; Li et al. [53]: before pregnancy group, OR = 0.67, 95% CI: 0.56–0.80, *I*^2^ = 87.20%, *p* < 0.001; Braun et al. [56]: 4-year group, OR = 0.67, 95% CI: 0.56–0.80, *I*^2^ = 86.70%, *p* < 0.001) yielded results consistent with the primary analysis. Similarly, replacing the AD subtype from Surén et al. [39] with the AS subtype yielded a pooled effect size of OR = 0.66 (95% CI: 0.55–0.79, *I*^2^ = 86.90%, *p* < 0.001), while substitution with the PPD-NOS subtype produced an OR of 0.67 (95% CI: 0.56–0.81, *I*^2^ = 86.80%, *p* < 0.001). None of these modifications resulted in substantial changes to the overall meta-analytic findings. The pooled effect size from high-quality MAs of primary studies was an OR of 0.70 (95% CI: 0.58–0.85; *I*^2^ = 88.50%; *p* < 0.001), which aligns with the overall findings.

After excluding studies with high heterogeneity (*I*^2^ > 90.00%), including cohort studies, those with large sample sizes (>10,678), and those limited to supplementation during pregnancy only, the results (OR = 0.57, 95% CI: 0.47–0.70, *I*^2^ = 33.90%, *p* = 0.169) continued to demonstrate a protective effect of maternal folic acid supplementation during the periconceptional or early pregnancy period on the risk of offspring ASD. Although the Q-test was not statistically significant, the *I*^2^ value indicated moderate heterogeneity. To ensure robustness, the DL random-effects model was used for pooling effect sizes. However, due to the limited number of included studies (n = 6), as well as the presence of publication bias and excessive significance bias (all *p* < 0.001), these results should be interpreted with caution.

#### 3.3.2. Motor Development

**Figure 4:**
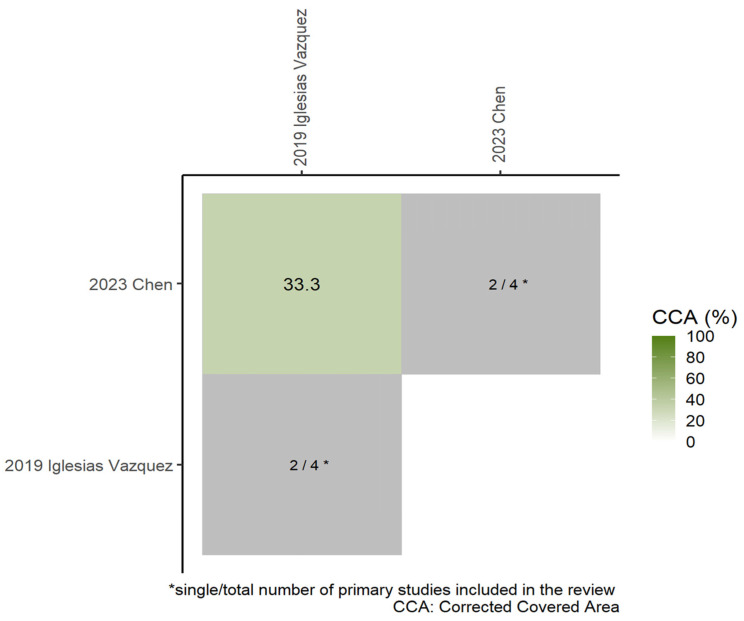
Heatmap of overlapping visualizations of original studies per pair of MAs (Motor Development). (*CCA* = 0% indicates no overlap of original studies [white] and *CCA* = 100% indicates complete overlap of original studies between meta [dark green]. The gray diagonal tiles present the single/total number of primary studies that included in each review.) [12,15].

Two MAs investigated the association between maternal folic acid supplementation and offspring motor development outcomes. Chen et al. [12] reported a potential positive association, while Vazque et al. [15] observed a slight negative association; however, neither association was statistically significant. The original studies included in these MAs showed substantial overlap (*CCA* = 33.3%). Figure 4 illustrates the extent of overlap in original studies between each pair of MAs. Based on the predetermined selection criteria, the MA by Chen et al. [12] was selected for primary data extraction and was supplemented with original studies not included therein. The final data extraction results are provided in Supplementary Materials, Table S11.

Although multiple effect sizes are present in the original studies by Chatzi et al. [60] and Valera-Gran et al. [61], they could not be combined due to their mutual independence. Specifically, Chatzi et al. [60] categorized folic acid supplementation doses of 1000–5000 μg/d and >5000 μg/d as separate exposure groups, comparing each to the non-supplemented group to obtain independent effect estimates. Similarly, Valera-Gran et al. [61] provided independent effect size data for inclusion in the MA by Vazquez et al. [15]. Consequently, these effect sizes were directly incorporated into the main analysis without selection.

**Figure 5:**
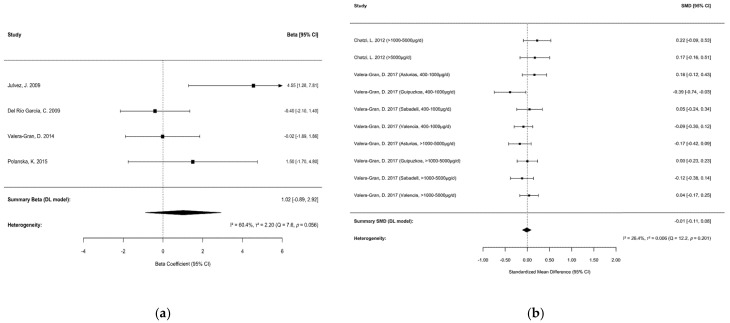
Forest plot showing the association between maternal folic acid supplementation and Motor Development in offspring: (**a**) psychomotor development (Beta); (**b**) motor development (SMD). Individual study estimates are denoted by squares, with horizontal lines representing 95% CIs; arrows indicate truncated CIs. The diamond in each plot represents the pooled effect size, with its center and width corresponding to the point estimate and 95% CI, respectively. Statistical significance is determined by the position of the diamond relative to the vertical null line (Beta/SMD = 0) [60,61,62,63,64,65].

Analysis using the DL random-effects model (R version 4.3.1; metafor package) indicated that maternal folic acid supplementation during pregnancy was not significantly associated with offspring psychomotor development (*β* = 1.02, 95% CI: −0.89 to 2.92; *I*^2^ = 60.40%, *p* = 0.056), as presented in Figure 5a. Sensitivity analysis employing the leave-one-out method showed that the pooled effect size ranged from *β* = 0.01 to 1.77, with consistent direction and no change in statistical significance. Exclusion of the study by Julvez et al. [62] yielded *β* = 0.01 with no heterogeneity, suggesting that this study may be a source of heterogeneity; however, the limited number of studies may constrain the statistical power of these analyses.

The SMD between the two groups estimated via the DL random-effects model was −0.01 (95% CI: −0.11 to 0.08; *I*^2^ = 26.4%, *p* = 0.201), suggesting no significant effect of prenatal folic acid supplementation on offspring motor development (Figure 5b). Sensitivity analyses using the leave-one-out method showed that exclusion of individual studies did not alter the non-significant results (SMD: −0.03 to 0.01; all 95% CIs included 0). Exclusion of the study by Valera-Gran et al. [61] (2017, Guipuzkoa, 400–1000 μg/d) substantially reduced heterogeneity (*I*^2^ = 0.0%, *p* = 0.465), indicating that this study may be a key source of between-study heterogeneity.

#### 3.3.3. ADHD, Mental Development, Language, Behavior, Intellectual or Cognitive Development

In this umbrella review, only 1 MA was identified for each of the following outcomes: ADHD, mental development, language, behavior, and intellectual or cognitive development. Among these, only the outcome for mental development was reported by Vazquez et al. [15], while the remaining outcomes were all derived from a single MA by Chen et al. [12].

The reanalysis of pooled effect sizes using the DL random-effects model is summarized in Supplementary Materials, Table S8. For ADHD, the pooled effect size (6 studies; OR = 0.86, 95% CI: 0.78–0.95; *I*^2^ = 0.0%, *p* = 0.678) is consistent with the original fixed-effects MA. No evidence of publication bias (Egger’s *p* = 0.74) or excess significance bias (O/E = 0.579, *p* = 0.265) was detected, supporting a protective effect of maternal folic acid supplementation against offspring ADHD. Similarly, the result for behavioral problems (OR = 0.75, 95% CI: 0.63–0.91; *I*^2^ = 0.0%, *p* = 0.520) aligns with the original fixed-effects estimate, also suggesting a protective effect. However, potential publication bias was indicated by Egger’s test (*p* = 0.026), a result that requires cautious interpretation given the limited number of studies (n = 3) for this outcome.

The reanalysis found that the pooled effect sizes for Language Problems (4 studies; *β* = 0.78, 95% CI: −1.17 to 2.72; *I*^2^ = 63.09%, *p* = 0.043) and Intellectual or Cognitive Development (5 studies; *β* = 1.30, 95% CI: −1.61 to 4.21; *I*^2^ = 72.64%, *p* = 0.006) were consistent with the original MA. No significant association was observed between maternal folic acid supplementation and either outcome. However, these results should be interpreted with caution due to the limited number of studies and considerable heterogeneity.

Vazquez et al. [15] identified 7 original studies [60,61,64,65,66,67,68] investigating mental development outcomes, with detailed information available in Supplementary Materials, Table S12. The included studies comprised 5 cohort studies and 2 RCTs. When data were aggregated using the DL random-effects model without accounting for study independence (7 studies; SMD = −0.06, 95% CI: −0.116 to 0.002; *I*^2^ = 35.55%, *p* = 0.036), the results were consistent with the original MA, indicating no significant effect of maternal folic acid supplementation on offspring mental development. A separate meta-analysis of cohort studies showed reduced heterogeneity (*I*^2^ = 26.42%, *p* = 0.112), though no significant association was observed. To avoid violating the independence assumption, the analysis used data from the longest follow-up duration to ensure outcome stability. Subsequent analyses continued to show no significant effect of supplementation on mental development, whether including all studies (SMD = −0.05, 95% CI: −0.14 to 0.04) or only cohort studies (SMD = −0.02, 95% CI: −0.09 to 0.06). Moreover, heterogeneity among cohort studies was further reduced to *I*^2^ = 0.0%, underscoring the robustness and stability of these findings.

### 3.4. Systematic Review

As presented in Table 2, among the included SRs and MAs, 13 reviews did not conduct meta-analyses due to concerns such as heterogeneity (excluding updated reviews) and provided only qualitative conclusions. Li et al. [32] performed an MA solely on ASD, whereas for ADHD, only a qualitative analysis was conducted owing to heterogeneity and related concerns. In total, 9 reviews reported outcomes related to ASD, and 4 addressed ADHD. Among these, 3 reviews focused on intelligence and cognitive function, motor function, language development, behavioral and emotional problems, and mental development, while only 1 review specifically evaluated broader neurodevelopmental outcomes.

**Figure 6:**
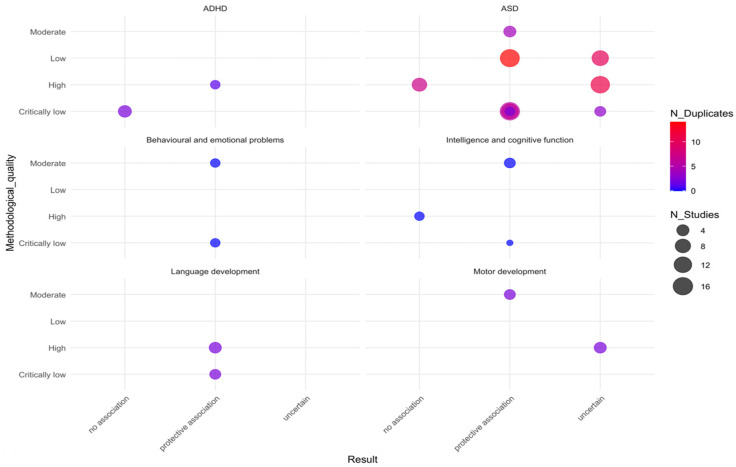
Bubble chart showing the distribution of outcomes in the SR.

In the context of ADHD, the reviews by Chmielewska et al. [75] and Gao et al. [13] are cited. Regarding motor development, language development, and behavioral and emotional problems, Chmielewska et al. [75] was excluded from qualitative synthesis because only one original study was available per outcome, and these studies overlapped with original research included in other reviews addressing the same outcomes. The remaining SRs incorporated relevant non-randomized studies pertaining to the topic, as summarized in Figure 6.

ASD is the most frequently studied outcome; however, only 3 SRs on this topic were rated as having moderate or higher methodological quality. Among the 9 reviews analyzed, 5 reported a protective effect, 1 found no association, and the remaining 4 were inconclusive. Although a relatively large number of original studies—covering broad populations—have been included for this outcome, significant overlap exists among them. Most inconclusive reviews failed to reach definitive conclusions due to variations in study populations, folic acid supplementation dosages, and timing windows.

3 SRs examined outcomes related to intelligence and cognitive function, with no overlap in the original studies included. Two of these reviews—focusing on Asian and Greek populations—suggest that preconception and/or prenatal folic acid supplementation has a protective effect on offspring intelligence and cognitive function. In contrast, the third review, which primarily involved American populations, found no association. Notably, the AMSTAR-2 quality ratings were higher for the review reporting no association than for those indicating a protective effect. Additionally, a double-blind randomized trial by Chimeh et al. [76], not included in the qualitative synthesis, also demonstrated a beneficial effect of maternal folic acid supplementation.

Two SRs addressed ADHD-related outcomes; however, there was considerable overlap in the original studies included, with a predominant focus on European populations. The review containing a larger number of original studies found no association between maternal folic acid supplementation and the incidence of ADHD in offspring; however, its methodological quality was rated as very low. Another review, which had a high AMSTAR-2 score, suggested a potential protective effect of supplementation, but this conclusion is limited by the inclusion of only two original studies and should be interpreted with caution.

Despite AMSTAR-2 ratings of “Moderate” and “CL”, respectively, both reviews consistently indicated that maternal folic acid supplementation before and/or during pregnancy may confer a protective effect against offspring behavioral and emotional problems. Although there was no overlap in the original studies between the two reviews addressing this outcome, and despite the inclusion of a prospective RCT published in 1991 (cited in Freedman et al. [77]) that was not qualitatively discussed, caution is still warranted when extrapolating this protective effect due to limitations in study quantity, publication date, and population characteristics.

Two SRs examined outcomes related to language development, primarily involving European and American populations, with substantial overlap in their original studies. Despite receiving AMSTAR-2 ratings of “High” and “CL”, respectively, both reviews consistently indicated that maternal folic acid supplementation before and/or during pregnancy may reduce the risk of language development delays in offspring.

Two SRs addressed outcomes related to motor development; however, the original studies exhibited substantial overlap, with research populations primarily concentrated in Europe. The methodological quality of these reviews was rated as “High” and “Moderate”, respectively. Sargoor et al. [23], which received a “High” rating, suggested that maternal folic acid supplementation before and/or during pregnancy may positively influence offspring motor development, though no definitive association could be established. Another review by Gao et al. [13]—which included an RCT not incorporated in the qualitative synthesis—also demonstrated a protective effect. Overall, no adverse effects were associated with routine-dose supplementation.

Although two SRs included outcomes related to mental development, only one relevant original study by Sargoor et al. [23] was identified, which also overlapped with Gao et al. [13]. This study suggested that folic acid supplementation at doses below 400 mg/day during early pregnancy may provide mental development benefits specifically for offspring of mothers with the TT genotype. In contrast, an RCT from the 1990s, cited in Gao et al. [13], found no significant association between folic acid intake and mental development. Chamova et al. [78]—rated as “CL” on AMSTAR-2—evaluated overall neurodevelopment using a Chinese birth cohort and reported that routine maternal folic acid supplementation (400 μg/day) during pregnancy was associated with improved neurodevelopment at one month of age. Given the limited number of studies and restricted population diversity, these findings require further validation through more extensive research.

## 4. Discussion

### 4.1. Main Finding

This umbrella review systematically evaluates existing SRs and MAs on the association between maternal folic acid supplementation during preconception and/or pregnancy and the risk of NDDs in offspring. The findings reveal that the overall methodological quality and level of evidence in the current literature are generally low. Based on MAs, maternal folic acid supplementation appears to be associated with a reduced risk of ASD, ADHD, and behavioral problems in offspring. However, no significant protective effects were observed for motor development, language problems, or intellectual, cognitive, and mental development. In qualitative reviews without meta-analysis, the results remain inconsistent; aside from behavioral and emotional problems and language development, where conclusions tend to suggest a protective association, findings for other outcomes are distributed among “protective association,” “no association,” and “uncertain association.”

### 4.2. Discussion on ASD

This umbrella review suggests that maternal folic acid supplementation during preconception and/or pregnancy may demonstrate a protective association against ASD in offspring. This protective effect appears more consistently observed when supplementation occurs during the periconceptional or early pregnancy periods.

A meta-analysis of the included studies indicated that maternal folic acid supplementation before and/or during pregnancy may reduce the risk of ASD in offspring. These findings align with those reported by Wang et al. [11]. Although the primary analyses showed substantial heterogeneity and potential publication bias, and the number of studies showing positive effects was greater than expected, subgroup and sensitivity analyses supported the stability of this protective association. The high heterogeneity observed in cohort studies, large-sample studies, and investigations of prenatal folic acid supplementation may be explained by variations in outcome assessment methods and insufficient documentation of supplementation initiation and duration. After excluding these studies, maternal folic acid supplementation during the periconceptional or early pregnancy periods demonstrated a more consistent and reliable protective effect against ASD in offspring.

In SRs without MA, most studies suggest a potential protective effect of maternal folic acid supplementation. Among these, although Sampaio et al. [69] was limited by both low methodological quality and low evidence strength, it incorporated the largest number of original studies and suggested that maternal folic acid supplementation during preconception and early pregnancy may confer a protective effect against ASD—a finding consistent with the sensitivity analyses mentioned above. Reviews that did not establish a definitive association generally reported no adverse effect of moderate folic acid supplementation on offspring ASD risk. For example, Cheng et al. [74] suggests the potential existence of an optimal folic acid intake level during pregnancy that may lower the risk of ASD.

Emerging evidence suggests that maternal folic acid supplementation may influence the etiology of offspring autism through mechanisms involving DNA methylation regulation, neurotransmitter synthesis and metabolism, and cerebrospinal fluid folate levels. For instance, folate deficiency may lead to aberrant DNA methylation patterns, thereby disrupting normal neuronal development; it may also impair neurotransmitter synthesis, affecting neuronal connectivity and function [79], ultimately increasing autism risk. In this umbrella review, the methodological quality of the included MAs (High: 50.00%) and the overall strength of evidence (Moderate/Low: 50.00%) were higher than those of the SRs (High/Moderate [Methodological quality]: 33.33%; Low [Evidence quality]: 22.22%). Furthermore, the overlap of primary studies among SRs ranged from 37.50% to 100%, with most reviews failing to report whether study protocols were registered a priori. These findings underscore the need for standardized reporting protocols in future research to minimize bias introduced by the repeated inclusion of identical studies across multiple SRs. In conclusion, this review supports the hypothesis that maternal folic acid supplementation before and/or during pregnancy may confer a protective effect against offspring ASD.

### 4.3. Discussion on ADHD

This umbrella review suggests that maternal folic acid supplementation during preconception and/or pregnancy may be associated with a reduced risk of ADHD in offspring.

This MA indicates a potential protective role of maternal folic acid supplementation against offspring ADHD. This finding is consistent with the results of the SR by Sargoor et al. [23]. Although SRs without meta-analysis—including that by Sargoor et al. [23]—were limited to only two studies, and their findings were inconsistent. In contrast, Li et al. [53] could not identify a significant protective association between folic acid supplementation and ADHD risk, though no adverse effects were reported. A comparison of the original studies included in our meta-analysis with those in Li et al. [53] revealed that the former incorporated more recent publications and, despite comparable evidence quality, employed more rigorous methodological approaches. Overall, these results strengthen the contention that maternal folic acid supplementation may help reduce the risk of ADHD in offspring. Moreover, several mechanistic studies suggest that gestational folate deficiency may contribute to impaired neuronal differentiation and disrupted neurotransmitter synthesis, potentially elevating ADHD risk. Additionally, maternal folic acid supplementation may modulate offspring gene expression related to ADHD through alterations in hepatic DNA methylation patterns [80].

Current SRs and MAs investigating the association between maternal folic acid supplementation and ADHD-related neurodevelopmental outcomes remain limited. Although the two available SRs present conflicting conclusions, one is consistent with the present meta-analytic findings. Moreover, our quantitative synthesis provides more robust evidence supporting a protective relationship between maternal folic acid intake and reduced risk of ADHD, thereby corroborating evidence from basic mechanistic studies. Further high-quality epidemiological research is needed to confirm this association and to establish more conclusive evidence regarding the potential protective role of folic acid supplementation in ADHD.

### 4.4. Discussion on Motor Development

Based on the current umbrella review, evidence regarding the association between maternal folic acid supplementation and offspring motor development remains inconclusive.

In this umbrella review, although the two MAs on motor development outcomes employed different effect size metrics, their overall conclusions consistently indicated no significant association between maternal folic acid supplementation (during preconception or prenatal periods) and offspring motor development, with stable results across analyses. These findings contrast with those reported by Bhate et al. [81,82]. Furthermore, the primary studies included in SRs without meta-analyses showed high overlap rates—50.00% and 66.67%, respectively—yet yielded inconsistent conclusions: one review described the relationship as “uncertain,” while the other suggested a “protective association.” These discrepancies are likely attributable to variations in outcome measurement tools, including differences between diagnostic assessments for motor development disorders and quantitative scoring of motor function.

The methodological quality (High/Moderate) and evidence quality (Low/Very Low) of SRs focusing on motor development as an outcome of neurodevelopmental disorders are generally higher than those of MAs (methodological quality: High/Low; evidence quality: Very Low). In summary, existing evidence cannot support a definitive conclusion regarding the association between maternal folic acid supplementation and offspring motor development. Importantly, routine-dose folic acid supplementation may not be linked to adverse effects. Future studies should prioritize standardized measurement and diagnostic criteria to facilitate more robust evaluation of the relationship between maternal folic acid supplementation during preconception and/or pregnancy and offspring motor development.

### 4.5. Discussion on Intellectual or Cognitive Development

In this umbrella review, the association between maternal folic acid supplementation during preconception and/or pregnancy and offspring intellectual or cognitive development remains inconclusive. As existing studies frequently combine these developmental domains in their analyses, precluding independent assessment, they will be discussed collectively as a single module in this review.

Among the MAs included in this umbrella review, only one investigated the association between maternal folic acid supplementation and offspring intellectual or cognitive development, and no significant relationship was identified. In contrast, among SRs, two-thirds of studies suggested a protective effect of preconception and/or prenatal folic acid supplementation on offspring intellectual or cognitive development, although this effect appears limited to Asian and Greek populations. Within this umbrella review, the evidence quality supporting “no association” (Low/Very Low) is marginally higher than that for a “protective association.” These discrepant conclusions are likely due to variations in sample sizes, study populations, and outcome assessment methodologies.

A long-term follow-up study (11 years) based on an RCT demonstrated that children of mothers who received folic acid supplementation during pregnancy achieved higher scores on cognitive tests at age 3 and showed significantly better performance on vocabulary and reasoning tests at age 7 compared to those in the placebo group [83]. Further research suggests that folic acid supplementation may enhance offspring cognitive development via epigenetic mechanisms, specifically DNA methylation of genes such as IGF2 [84]. However, due to inconsistencies across existing studies, the current evidence remains insufficient to establish a definitive relationship between maternal folic acid supplementation before or during pregnancy and offspring intellectual or cognitive development. Thus, further high-quality studies employing more rigorous methodologies are needed to clarify this association.

### 4.6. Discussion on Behavioral and Emotional Problems

In some studies on NDDs, behavioral and emotional problems are included as one of the outcomes in research and discussion. According to the umbrella review, maternal folic acid supplementation before and/or during pregnancy may have a protective effect against the occurrence of behavioral and emotional problems in offspring.

In this umbrella review, only one MA investigated behavioral problems as an outcome, suggesting a potential protective effect of maternal folic acid supplementation against this disorder in offspring, although potential publication bias cannot be excluded. Furthermore, two additional SRs—without meta-analyses—also assessed this outcome and similarly reported beneficial effects.

Current evidence suggests a protective association between the two; however, due to the overall low quality of evidence, limited number of studies, and restricted population coverage, further methodologically rigorous prospective studies—such as those minimizing reporting and recall bias—or RCTs are needed to strengthen the evidence base.

### 4.7. Discussion on Language Development

Currently, no definitive conclusion can be drawn regarding the association between maternal folic acid supplementation before and/or during pregnancy and the risk of offspring language development delays.

The MA included in this umbrella review (evidence quality: Very Low) found no association between maternal folic acid supplementation and offspring language development. In contrast, several SRs without meta-analysis—such as those by Chmielewska et al. [75] and Sargoor et al. [23] (evidence quality: Very Low/Moderate)—suggest a potential beneficial effect, though these are based on a limited number of primary studies. The discrepancy in findings may be attributable to differences in the studied populations and language assessment methods. The SRs primarily included European and North American populations and used screening-oriented tools such as the PPVT-III, DDST, questionnaires, and Bayley scales, which cover a relatively narrow scope of language domains. The MA, however, involved predominantly European and Oceanian populations and employed more comprehensive assessments, including the Bayley scales, CDI, and MCSA, which evaluate comprehension, expression, and language function across multiple contexts. Future research should prioritize the use of standardized assessment tools and conduct high-quality studies with more diverse population representation.

Although some lower-quality studies suggest that maternal folic acid supplementation may improve offspring language development, an MA—representing a higher level of evidence despite its very low certainty—showed no significant association. Given the overall very low quality of evidence and the presence of conflicting findings, no definitive conclusions can be drawn. Further high-quality studies are required to clarify whether any association exists.

### 4.8. Discussion on Mental Development and Neurodevelopmental Disorders

Current evidence could not permit definitive conclusions regarding the effects of maternal folic acid supplementation before and/or during pregnancy on offspring mental development. Available studies on neurodevelopmental outcomes remain limited in both quantity and scope.

Mental development in this context encompasses conditions such as anxiety disorders, depression, and neurodevelopmental disorders [1]. A 2019 MA by Vazquez et al. [15] found no association between maternal folic acid supplementation and offspring mental development—a finding consistently supported in subsequent re-analyses. While some SRs without meta-analysis suggest a potential protective effect, each was based on only a single original study, substantially limiting the robustness of their conclusions. Similarly, reviews focusing on neurodevelopmental outcomes also indicate a possible protective effect but rely exclusively on one relevant primary study.

Therefore, future research should prioritize larger sample sizes and more precise outcome classifications to better determine the effect size and direction of maternal folic acid supplementation on specific offspring neurodevelopmental outcomes.

### 4.9. Strength

As the highest form of evidence synthesis, this umbrella review systematically evaluates and integrates existing SRs and MAs on the association between maternal folic acid supplementation—before and/or during pregnancy—and the risk of various neurodevelopmental disorders in offspring. It offers both methodological and practical advantages by providing a comprehensive, high-level overview of the current evidence base. First, the review delivers a broad assessment of the entire research landscape, moving beyond the narrow scope of individual reviews by synthesizing evidence from multiple sources. This approach allows for the identification of consistencies, contradictions, and evidence gaps related to folic acid interventions across different neurodevelopmental outcomes, thereby supporting a more nuanced and thorough understanding of the field. Second, standardized tools, including AMSTAR-2 and GRADE, were used to evaluate the methodological quality of the included reviews and MAs and to assess the certainty of the evidence for key outcomes. This dual assessment provides clinicians and public health policymakers with a transparent and critical appraisal, clarifying which findings are most reliable and on what grounds, thereby facilitating evidence-informed decision-making. Furthermore, this work helps distinguish robust conclusions from persistent uncertainties and may help prevent unnecessary duplication of low-quality research by synthesizing and evaluating existing literature.

Overall, these advantages establish the present umbrella review as an authoritative reference that not only synthesizes the current state of knowledge, but also critically informs clinical and public health practice, while guiding future research on the association between maternal folic acid supplementation—before and/or during pregnancy—and the risk of distinct neurodevelopmental disorders in offspring.

### 4.10. Limitations

This umbrella review has several limitations. First, the methodological and evidence quality of existing SRs/MAs is generally low, and the inconsistency among research findings undermines the robustness of the conclusions drawn. Second, owing to ethical and practical constraints, there are relatively few RCTs in this area. Although our umbrella review included only non-randomized studies, our initial search did not restrict the study design. After extensive retrieval, only the original studies within the SR by Skórka et al. (2012) [85] included RCTs, with conclusions similar to those of our umbrella review; Third, most SRs/MAs did not report the dosage or duration of folic acid supplementation, which precludes dose–response analysis and allows only a rough estimation of the supplementation time window. Finally, heterogeneity in the diagnostic criteria for the disease may introduce measurement bias and complicate the comparability and synthesis of results. In summary, the current evidence remains far from conclusive. Future studies with more rigorous methodologies and higher quality are needed to strengthen the evidence base.

### 4.11. Implications of Findings

This review indicates that although evidence supporting maternal folic acid supplementation before and/or during pregnancy for the prevention of NDDs in offspring remains limited, the findings still carry substantial clinical and public health relevance. It is well-established globally that folic acid supplementation during the preconception period and early pregnancy effectively prevents NTDs. Our results suggest that, at standard recommended doses, folic acid supplementation could not exhibit clear adverse effects on any NDD outcomes, thereby reinforcing current public health strategies that advocate folic acid supplementation. Furthermore, the study suggests a potential protective role of folic acid in reducing the risk of ASD, ADHD, and behavioral and emotional problems. While higher-quality evidence is needed to confirm these benefits, these preliminary insights highlight promising avenues for early-life interventions aimed at preventing such conditions. These findings offer additional motivation for women of reproductive age to adhere to folic acid supplementation. Thus, clinicians and public health practitioners should continue to emphasize the benefits of folic acid for offspring health and recommend routine supplementation for all women who are planning pregnancy or are in early pregnancy, in accordance with established guidelines.

## 5. Conclusions

Maternal folic acid supplementation before and/or during pregnancy may reduce the risk of offspring developing ASD, ADHD, and behavioral or emotional problems. The most consistent protective association was observed for ASD with supplementation during the periconceptional or early pregnancy periods. However, the overall quality of evidence is low, and limited data preclude definitive conclusions regarding effects on motor, intellectual, cognitive, and language development. Ultimately, the key contribution of this review lies in delineating the current state of the evidence, highlighting its weaknesses, and thereby stimulating and informing the design of more rigorous primary studies to explicitly address remaining questions regarding dosage and duration, etc. Given the established benefits of folic acid in preventing NTDs and the absence of robust evidence for other adverse neurodevelopmental effects, we strongly recommend that women of reproductive age adhere to current supplementation guidelines.

## Figures and Tables

**Table 2:** Characteristics of included systematic reviews.

Author (year)	Exposure		Exposure Comparison	Assessment Criteria/Tool	Study Design (Number of Studies)	No. of Participants (Cases)	Databases Searched	Locations	Main Findings
	Dose	Window							
ASD									
Sampaio et al. [69]	NA	Preconception period and at the beginning of pregnancy	Not applicable or insufficient	NA	All: 17	NA	BIREME virtual bank, Virtual Health Library, the Medical Literature Analysis and Retrieval System Online (between February 2018 and February 2020)	The Netherlands, the United States, Denmark, China, Colombia, Norway, Palestine	Maternal FA supplementation in the pre-conception period and beginning of pregnancy as a protective effect in relation to ASD.
Hoxha et al. [70]	NA	Before and/or during pregnancy	No folic acid supplementation	PDD-NOS, MABC, etc.	All: 10 Cohort studies: 9 Case–control cohort study: 1	1,083,144 (NA)	PubMed, Scopus, Medline, and Embase (until 31 May 2021)	Denmark, Israel, Norway, Sweden, the United States, the Netherlands, Nepal	In some of them, the maternal FA supplementation results in a reduced ASD risk; other studies do not confirm these positive results, finding an enhanced risk following the supplementation; furthermore, some authors did not obtain any association between folate intake and ASD risk, or not satisfactory conclusions about the utility of folate supplementation.
Gao et al. [13]	NA	Before and/or during pregnancy	No folic acid supplementation	ADI-R, ADOS, etc.	About: 4 Cohort studies: 2 Case–control studies: 2	90,613 (NA)	Medline, EMBASE (until 31 December 2014)	Norway, the United States, and Spain	Folic acid supplementation in pregnancy may protect against impaired neurodevelopment, including ASDs in children.
Castro et al. [22]	NA	Perinatal period and/or pregnancy	No folic acid supplementation	ADI-R, ADOS	About: 2 Cohort study: 1 Case–control study: 1	86,013 (829)	MEDLIN (from January 2003 to July 2013)	Norway, the United States	Periconceptional folic acid may reduce ASD risk.
DeVilbiss et al. [71]	NA	pre- and peri-conceptional periods	No folic acid supplementation	SRS, ADI-R, etc.	All: 10 Cohort studies: 9 Case–control study: 1	138,100 (NA)	PubMed (until 15 April 2015)	Norway, the United States, The Netherlands, Spain, India, Nepal, and Hungary	Observational studies: Maternal FA intake during the periconceptional period and early pregnancy has a protective effect on the risk of ASD in children. RCT: Maternal folic acid supplementation may have a positive effect on autism in offspring, or it may be unrelated.
Zhong et al. [72]	NA	Perinatal period and/or pregnancy	With lower/lowest levels as the referent group	DSM-IV, ADOS, MSEL, ADI-R, SCQ, SRS	About: 15 Cohort studies: 9 Case–control studies: 4 nested case–control studies: 2	661,282 (NA)	PubMed (until July 2020)	Norway, the United States, Israel, the Netherlands, Denmark, Sweden, China	Higher or moderate intake of prenatal FA was associated with reductions in odds of ASD, though results have not been uniform, and there is a need to clarify differences in findings based on biomarkers versus reported intake.
Viswanathan et al. [73]	NA	Perinatal period and/or pregnancy	No folic acid supplementation	ICD-10, DSM-IV, etc.	About: 7 Cohort studies: 6 Case–control study: 1	761,125 (NA)	PubMed, Cochrane Library, Embase, and trial registries (from 1 July 2015, through 2 July 2021, with surveillance through 10 February 2023)	Israel, Denmark, Sweden, Norway	There is no association between folic acid supplementation before or during pregnancy and the risk of autism in offspring.
Vasconcelos et al. [16]	NA	Perinatal period and/or pregnancy	No folic acid supplementation	DSM-5, ICD-9/10	About: 14 Case–control studies: 13 Cross-sectional studies: 4 Both: 3	69,484 (5085)	PubMed (MEDLINE), EBSCO, and CINAHL databases (from 2013 to 2024)	The United States, Sweden, Israel, China	An association between the risk of ASD and maternal folic acid intake was not established by most studies included in this review. Further investigation is needed to define a causal relationship between maternal folic acid intake and the risk of autism.
Cheng et al. [74]	NA	During the perinatal period or pregnancy	No folic acid supplementation	ICD, ADI-R, ADOS	About: 3 Prospective Cohort study: 1 Case–control studies: 2	2700 (NA)	MEDLINE (between 1 January 2005 and 1 July 2018)	The United States	There may be an optimal level of folate during pregnancy for reducing the offspring ASD risk.
Chmielewska et al. [75]	NA	Periconceptional period	No folic acid supplementation	ADI-R, ADOS, expert diagnosis	About: 2 Cohort study: 1 case–control study: 1	86,013 (NA)	Cochrane Library (2009–May 2014)	The United States, Norway	Maternal folic acid intake during pregnancy is associated with a lower risk of ASD in offspring.
ADHD									
Li et al. [32]	NA	Periconception period, early pregnancy	No folic acid supplementation and/or normal diet	ICD-10, SDQ, patient registries, ADHD Rating Scale-IV	All: 5 Cohort studies: 5	43,063 (NA)	PubMed and Embase (through March 2019)	New Zealand, Japan, Denmark, Spain, United Kingdom	No convincing evidence supports an association between folate intake from food or supplements and ADHD risk.
Gao et al. [13]	NA	Periconception period, early pregnancy	No folic acid supplementation	NA	About: 1 Cohort study: 1	393 (NA)	Medline, EMBASE (until 31 December 2014)	Norway, the United States, Spain	Omission errors were lower in those children whose mothers took dietary supplementation with folic acid during pregnancy.
Sargoor et al. [23]	NA	Periconception period, early pregnancy	No folic acid supplementation	By two psychologists and teachers, CPT-II	About: 2 Cohort studies: 2	813 (NA)	Medline, PubMed, and the Cochrane Library (from January 1960 to October2014)	Spain, the United Kingdom	Folic acid supplementation during pregnancy may have beneficial effects on symptoms of inattention and hyperactivity/impulsivity.
Chmielewska et al. [75]	NA	Periconception period, early pregnancy	No folic acid supplementation	SDQ	About: 1 prospective cohort study: 1	100 (NA)	MEDLINE, the Cochrane Library (2009–May 2014)	The United Kingdom	Higher total folate intake from food and supplements during early pregnancy is associated with higher rates of attention deficit hyperactivity disorder in children.
Motor Development									
Gao et al. [13]	NA	Before and/or during pregnancy	No folic acid supplementation	MABC, BSID, etc.	About: 4 RCT: 1 Cohort studies: 3	10,083 (NA)	Medline, EMBASE (until 31 December 2014)	Nepal, Spain	Folic acid supplementation in pregnancy may improve motor function.
Sargoor et al. [23]	NA	Before and/or during pregnancy	No folic acid supplementation	DDST, Questionnaire, etc.	About: 4 Prospective observational study: 1 Population-based longitudinal study: 1 Cohort studies: 2	46,244 (NA)	Medline/PubMed and the Cochrane Library (from January 1960 to October 2014)	The United Kingdom, Spain, Norway, and the United States	Half of the studies showed no association between folic acid supplementation and motor development, while half demonstrated a protective effect.
Chmielewska et al. [75]	NA	Periconceptional period	No folic acid supplementation	Denver development scale	About: 1 Population-based longitudinal study: 1	6774 (NA)	MEDLINE, the Cochrane Library (2009–May 2014)	The United States	Folic acid use was associated with improved gross motor development.
Intellectual or cognitive development									
Gao et al. [13]	NA	Before and/or during pregnancy	No folic acid supplementation	WISC-III, K-ABC, etc.	All: 3Cohort studies: 3	1900 (NA)	Medline, EMBASE (until 31 December 2014)	Nepal, India	Folic acid supplementation in pregnancy may improve intellectual and cognitive function.
Chimeh et al. [76]	NA	Perinatal period and/or pregnancy	No folic acid supplementation	BSID-III, Cognitive, linguistic, motor, socio-emotional, and adaptive behavioral subscales, MDI	About: 2 Prospective cohort study: 1 Two-way randomized trial:1	908 (NA)	Scopus, SID, Google Scholar, PubMed, and Science Direct (until March 2022)	Greece, China	Taking folic acid supplements can improve cognitive function in young children.
Sargoor et al. [23]	NA	Early pregnancy and mid-pregnancy	No folic acid supplementation	WRAML2, PPVT-III	About: 2 Prospective observational study: 1 Cohort study: 1	2105 (NA)	Medline/PubMed and the Cochrane Library (from January 1960 to October2014)	The United States	No association of folate intake with cognitive function.
Behavioral and emotional problems									
Gao et al. [13]	NA	Before and/or during pregnancy	No folic acid supplementation	CBCL 11/2–5	About: 2 Cohort studies: 2	7504 (NA)	Medline, EMBASE (until 31 December 2014)	Netherlands	Folic acid supplementation in pregnancy may improve behavioral and emotional problems.
Freedman et al. [77]	NA	Perinatal period and/or pregnancy	No folic acid supplementation	ANT, CBCL	About: 3 Prospective, randomized study: 1 Prospective observational studies: 2	3521 (NA)	EDLINE (from 1990 through 2017)	Europe, Netherlands	Decreased emotional problems (child).
Chmielewska et al. [75]	NA	Early pregnancy	No folic acid supplementation	Child Behavior Checklist	About: 1 Cohort study: 1	4214 (NA)	Cochrane Library (2009–May 2014)	The Netherlands	Children of mothers who did not use folic acid supplements during early pregnancy had a higher risk of developing problems.
Language development									
Gao et al. [13]	NA	Before and/or during pregnancy	No folic acid supplementation	Language grammar rating scale	About: 1 Cohort study: 1	38,954 (NA)	Medline, EMBASE (until 31 December 2014)	Norway	Folic acid supplementation in pregnancy may improve language function.
Sargoor et al. [23]	NA	Before and/or during pregnancy	No folic acid supplementation	PPVT-III, DDST, Questionnaire	About: 4 Prospective observational studies: 2 Cohort studies: 2	40,680 (NA)	Medline/PubMed and the Cochrane Library (from January 1960 to October 2014)	The United States, Norway, the United Kingdom, Spain	Most opinions support the potential benefits of folic acid for language function.
Chmielewska et al. [75]	NA	Before and/or during pregnancy	No folic acid supplementation	PPVT-III, Bayley	About: 3 prospective observational study: 1 cohort studies: 2	40,717 (NA)	MEDLINE, the Cochrane Library (2009–May 2014)	The United States, Norway, Greece	Early pregnancy use of folic acid supplements is associated with a reduced risk of language development delays in offspring.
Mental development									
Gao et al. [13]	NA	Before and/or during pregnancy	No folic acid supplementation	Bayley test	About: 2 RCT: 1 Cohort study: 1	878 (NA)	Medline, EMBASE (until 31 December 2014)	Hungary, Mexico	Folic acid supplementation in pregnancy may improve mental development in children.
Sargoor et al. [23]	Deficient daily folate intake (≥400 μg/d)	First trimester	Deficient daily folate intake (<400 μg/d)	Bayley test	About: 1 (Prospective birth cohort)	253 (NA)	Medline/PubMed and the Cochrane Library (from January 1960 to October 2014)	Mexico	Dietary intake of folate (<400 mg/day) reduced the mental development index only among children of mothers who were carriers of the TT genotype.
					Neurodevelopment				
Chamova et al. [78]	400 μg/d	Users who used it for 1–3 months, 3–6 months, and >6 months during pregnancy	Did not use folic acid supplements during pregnancy	Standard neuropsychological examination table	About: 1 Birth Cohort study: 1	1186 (NA)	PubMed, Scopus, Mendeley (2018–2023)	China	Maternal FA supplementation during pregnancy favors neurodevelopment in the offspring at 1 month old.

NA = Not Available; About = Only original studies pertinent to the research topic have been included; FA = Folic Acid; PDD-NOS = Pervasive developmental disorder-not otherwise specified; MABC = movement assessment battery for children; ADI-R = Autism Diagnostic Interview-Revised; ADOS (-G) = Autism Diagnostic Observation Schedule (-Generic); SRS = Social Responsiveness Scale; DSM-V/IV = Diagnostic and Statistical Manual of Mental Disorders (Fifth/Fourth Edition); MSEL = Mullen Scales of Early Learning; SCQ = Social Communication Questionnaire; ICD-8/9/10 = International Classification of Diseases-8/9/10; SDQ = Strengths and Difficulties Questionnaire; ADHD Rating Scale-IV = Attention-Deficit/Hyperactivity Disorder Rating Scale-Fourth Edition; CPT-II = Conners’ Continuous Performance Test-II; WISC-III = Wechsler Intelligence Scale for Children-Third Edition; K-ABC = Kaufman Assessment Battery for Children; BSID = Bayley Scales of Infant Development; MDI = Mental Developmental Index; WRAML2 = Wide Range Assessment of Memory and Learning -Second Edition; PPVT-III = Peabody Picture Vocabulary Test III; DDST = Denver developmental screening test; CBCL = Child Behavior Check List; ANT = Attention Network Test; EBSCO = Elton B. Stephens Company; SID = Scientific Information Database.

## Data Availability

All data used in this work are presented in the Supplementary Material and are available in the original publications.

## Supplementary Materials

The following supporting information can be downloaded at: https://www.mdpi.com/article/10.3390/nu17213443/s1, Tables S1–S5: Search strategies for each database; Table S6: AMSTAR-2 scores for outcomes of neurodevelopmental disorders in meta-analyses/systematic reviews; Table S7: GRADE ratings of meta-analyses/systematic reviews of neurodevelopmental disorders; Table S8: Results of heterogeneity (*I*^2^ and Q test) and publication bias (Egger test, over-significance test); Table S9: ASD original study information extraction table; Table S10: Subgroup analysis of original research on autism spectrum disorders (ASD); Table S11: Motor Development Original Study Information Extraction Table; Table S12: Mental development original study information extraction table.

## Abbreviations

The following abbreviations are used in this manuscript:

NDDs	Neurodevelopmental disorders
SRs	Systematic reviews
MAs	Meta-analyses
ASD	Autism spectrum disorder
ADHD	Attention-deficit/hyperactivity disorder
DSM-5	The *Diagnostic and Statistical Manual of Mental Disorders, Fifth Edition*
ICD-10/11	The 10th/11th revisions of the International Classification of Diseases
IDD	Intellectual Developmental Disorder
TDs	Tic disorders
NTDs	Neural tube defects
PROSPERO	The International Prospective Register of Systematic Reviews
CL	Critically low
GRADE	Grading of Recommendations, Assessment, Development, and Evaluations
RCTs	Randomized controlled trials
DL	DerSimonian and Laird
CIs	Confidence intervals
*CCA*	Corrected covered area
AD	Autism disorder
OR	Odds ratio
CI	Confidence interval
