# Supplementary material for: The Effect of Maternal Folic Acid Supplementation on Neurodevelopmental Disorders in Offspring: An Umbrella Review of Systematic Reviews and Meta-Analyses
Source: Nutrients. 2025 Oct 31;17(21):3443. doi: 10.3390/nu17213443 (PMC12608568; doi:10.3390/nu17213443)
Supplement: Supplementary file 1 [file nutrients-17-03443-s001.zip › nutrients-3910245-supplementary.pdf]

nutrients

MDPI

Supplementary Materials

Table S1. Search strategy for PubMed.
Table S2. Search strategy for Embase.
Table S3. Search strategy for The Cochrane Library.
Table S4. Search strategy for Web of Science.
Table S5. Search strategy for Medline (Ovid).
Table S6. AMSTAR-2 scores for outcomes of neurodevelopmental disorders in meta-analyses/systematic reviews.
Table S7. GRADE ratings of meta-analyses/systematic reviews of neurodevelopmental disorders.
Table S8. Results of heterogeneity (I^2 and Q test) and publication bias (Egger test, over-significance test).
Table S9. ASD original study information extraction table.
Table S10. Subgroup analysis of original research on autism spectrum disorders (ASD).
Table S11. Motor Development Original Study Information Extraction Table.
Table S12. Mental development original study information extraction table.

Table S1. Search strategy for PubMed (58 records, accessed in 2025/06/30).

No.	Description or domain	Search terms
1	MeSH terms for folic acid	"Folic Acid"[Mesh]
2	Search terms for folic acid [Title/Abstract]	"folate acid" OR "folate" OR "folate sodium" OR "folavit" OR "foldine" OR "foliamin" OR "folic*" OR "folin*" OR "folaci*" OR "folart" OR "folitab" OR "folium acid" OR "folivit" OR "folsa*" OR "folverlan" OR "folvite" OR "Vitamin B9" OR "Vitamin M" OR "acfol" OR "acide folique ccd" OR "acido folico" OR "acifolic" OR "apo folic" OR "gravi fol" OR "ingafol" OR "lactobacillus casei" OR "pteroylglutamic acid" OR "21179 34 0" OR "36653 55 1" OR "60672 17 5" OR "65165 91 5" OR "65165 92 6" OR "78168 16 8" OR "9p9w8ggu78"
3	MeSH terms for neurodevelopmental disorders	((((((((((((((((((("Neurodevelopmental Disorders"[Mesh]) OR "Autism Spectrum Disorder"[Mesh]) OR "Intellectual Disability"[Mesh]) OR "Communication Disorders"[Mesh]) OR "Language Disorders"[Mesh]) OR "Speech Sound Disorder"[Mesh]) OR "Childhood-Onset Fluency Disorder"[Mesh]) OR "Social Communication Disorder"[Mesh]) OR "Attention Deficit Disorder with Hyperactivity"[Mesh]) OR "Specific Learning Disorder"[Mesh]) OR "Dyslexia"[Mesh]) OR "Agraphia"[Mesh]) OR "Dyscalculia"[Mesh]) OR "Motor Disorders"[Mesh]) OR "Motor Skills Disorders"[Mesh]) OR "Stereotypic Movement Disorder"[Mesh]) OR "Tic Disorders"[Mesh]) OR "Tourette Syndrome"[Mesh]) OR "Child Development Disorders, Pervasive"[Mesh]) OR "Rett Syndrome"[Mesh]) OR "Asperger Syndrome"[Mesh]) OR "Learning Disabilities"[Mesh]
4	Search terms for neurodevelopmental disorders [Title/Abstract]	"neurodevelopmental disorder*" OR "mental retardation" OR "mental insufficiency" OR "mental symptom" OR "mental disorder*" OR "mental abnormality" OR "mental change" OR "mental confusion" OR "mental defect" OR "mental deficienc*" OR "mental disease" OR "mental disturbance" OR "mental health issue" OR "mental* ill*" OR "mental health problem" OR "nervous twitch" OR "neuropsychiatric disease*" OR "neuropsychiatric disorder*" OR "psychiatric illness" OR "psychiatric symptom" OR "psychi* disease*" OR "psychi* disorder*" OR "psychic disturbance" OR "psychologic* disorder" OR "psychologic* disturbance" OR "autis*" OR "ASD" OR "clumsy child* syndrome" OR "high functioning autism" OR "kanner syndrome" OR "intellectual disabilit*" OR "intellectual disorder*" OR "intellectual dysfunction" OR "IQ" OR "intellectual impairment" OR "Idiocy" OR "psychosocial mental retardation*" OR "ID" OR "IDD" OR "communicatio* disorder*" OR "communication disabilit*" OR

		"communication disease" OR "communication problem" OR "communicative dysfunction*" OR "agraphia*" OR "alexia*" OR "disfluency" OR "articulat* disorder*" OR "articulat* dysfunction" OR "articulat* impairment" OR "childhood onset fluency disorder*" OR "developmental apraxia" OR "fluency disorder" OR "mis articulation" OR "nonfluent speech" OR "phonetic disorder" OR "phonologic* impairment" OR "phonological disorder*" OR "speech disarticulation" OR "speech nonfluency" OR "speech sound disorder*" OR "stammering" OR "ADDH" OR "attention deficit disorder*" OR "overactive" OR "hyperactiv*" OR "ADHD" OR "ADD" OR "attention deficit and disruptive behavio*r disorders" OR "inattent*" OR "minimal brain dysfunction" OR "learning disabilit*" OR "learning disorder*" OR "learning disturbance*" OR "developmental academic disabilit*" OR "developmental academic disorder*" OR "learning deficit" OR "learning difficulty" OR "learning impairment" OR "learning problem" OR "impaired learning" OR "acalculia*" OR "developmental disabilities of scholastic skills" OR "developmental disorders of scholastic skills" OR "primary dyscalculias" OR "scholastic skills development disorders" OR "word blindness*" OR "handwriting disorder" OR "math* disability" OR "mathematics disorder" OR "writing difficulty" OR "strephosymbolia" OR "secondary acalculia*" OR "dyscalculia*" OR "dysgraphia*" OR "dyslectic child" OR "dyslexia*" OR "developmental dyspraxia" OR "habit spasm" OR "motor disability" OR "motor disfunction" OR "motor disorders" OR "motor disturbance" OR "motor dysfunction" OR "motor impairment" OR "motor skill* disorder*" OR "developmental coordination disorder*" OR "body rocking" OR "stereotypic movement disorder*" OR "tic disorder*" OR "nervous tic" OR "tourette* disease" OR "tourette* disorder" OR "tourette* syndrome" OR "pervasive and specific developmental disorders" OR "pervasive development* disorder*" OR "disintegrative disorder" OR "PDD" OR "rett* disorder" OR "rett* syndrome" OR "morbus rett" OR "rett disease" OR "asperger* disease*" OR "asperger* disorder*" OR "asperger* syndrome" OR "developmental speech or language disorders" OR "reading problem" OR "reading error" OR "reading disturbance" OR "reading disorder*" OR "reading disabilit*" OR "reading difficulty" OR "developmental learning disorders" OR "hyperkinet*" OR "language disorder*" OR "language deficiency" OR "language impairment" OR "language disability" OR "central language imbalance" OR "development* delay*" OR "stuttering" OR "opposition* defiant disorder*" OR "conduct disorder*"
5	MeSH terms for Intervention population	((((("Mothers"[Mesh]) OR "Maternal Exposure"[Mesh]) OR "Pregnant People"[Mesh]) OR "Pregnancy"[Mesh]) OR "Preconception Care"[Mesh]) OR "Gravidity"[Mesh]
6	Search terms for Intervention population [Title/Abstract]	"childbearing" OR "gestation" OR "gravidit*" OR "labo*r presentation" OR "maternal exposure*" OR "mother*" OR "multigravidit*" OR "nulligravidit*" OR "preconception care" OR "pregnanc*" OR "pregnant people*" OR "pregnant person" OR "pregnant wom*n" OR "prepregnancy care" OR "primigravidit*" OR "maternal" OR "prenatal" OR "antenatal" OR "per-conception*" OR "per-pregnancy" OR "per-implantation" OR "before pregnancy" OR "before conception"
7	MeSH terms for meta-analyses and systematic review	((("Meta-Analysis" [Publication Type]) OR "Meta-Analysis as Topic"[Mesh]) OR "Systematic Review" [Publication Type]) OR "Systematic Reviews as Topic"[Mesh]
8	Search terms for meta-analyses and systematic review [Title/Abstract]	"meta analy*" OR "Systematic Review" OR "Systematic Review* as Topic" OR "meta regression" OR "meta synthes*"
9	Total search string	(#1 OR #2) AND (#3 OR #4) AND (#5 OR #6) AND (#7 OR #8)

Table S2. Search strategy for Embase (209 records, accessed in 2025/06/30).

No.	Description or domain	Search terms
1	Emtree terms for folic acid	'folic acid'/exp
2	Search terms for folic acid (title/abstract/keywords)	'folate acid'2 OR 'folate' OR 'folate sodium' OR 'folavit' OR 'foldine' OR 'foliamin' OR 'folic*' OR 'folin*' OR 'folaci*' OR 'folart' OR 'folitab' OR 'folium acid' OR 'folivit' OR 'folsa*' OR 'folverlan' OR 'folvite' OR 'vitamin b9' OR 'vitamin m' OR 'acfol' OR 'acide folique ccd' OR 'acido folico' OR 'acifolic' OR 'apo folic' OR 'gravi fol' OR 'ingafol' OR 'lactobacillus casei' OR 'pteroylglutamic acid' OR '21179 34 0' OR '36653 55 1' OR '60672 17 5' OR '65165 91 5' OR '65165 92 6' OR '78168 16 8' OR '9p9w8ggu78'
3	Domain for folic acid	#1 OR #2
4	Emtree terms for neurodevelopmental disorders	'learning disorder'/exp OR 'asperger syndrome'/exp OR 'rett syndrome'/exp OR 'gilles de la tourette syndrome'/exp OR 'tic'/exp OR 'stereotypic movement disorder'/exp OR 'developmental coordination disorder'/exp OR 'motor dysfunction'/exp OR 'dyscalculia'/exp OR 'dysgraphia'/exp OR 'dyslexia'/exp OR 'learning disorder'/exp OR 'attention deficit hyperactivity disorder'/exp OR 'fluency disorder'/exp OR 'speech sound disorder'/exp OR 'language disability'/exp OR 'communication disorder'/exp OR 'intellectual impairment'/exp OR 'autism'/exp OR 'mental disease'/exp
5	Search terms for neurodevelopmental disorders (title/abstract/keywords)	'neurodevelopmental disorder*' OR 'mental retardation' OR 'mental insufficiency' OR 'mental symptom' OR 'mental disorder*' OR 'mental abnormality' OR 'mental change' OR 'mental confusion' OR 'mental defect' OR 'mental deficienc*' OR 'mental disease' OR 'mental disturbance' OR 'mental health issue' OR 'mental* ill*' OR 'mental health problem' OR 'nervous twitch' OR 'neuropsychiatric disease*' OR 'neuropsychiatric disorder*' OR 'psychiatric illness' OR 'psychiatric symptom' OR 'psychi*

		disease*' OR 'psychi* disorder*' OR 'psychic disturbance' OR 'psychologic* disorder' OR 'psychologic* disturbance' OR 'autis*' OR 'asd' OR 'clumsy child* syndrome' OR 'high functioning autism' OR 'kanner syndrome' OR 'intellectual disabilit*' OR 'intellectual disorder*' OR 'intellectual dysfunction' OR 'iq' OR 'intellectual impairment' OR 'idiocy' OR 'psychosocial mental retardation*' OR 'id' OR 'idd' OR 'communicatio* disorder*' OR 'communication disabilit*' OR 'communication disease' OR 'communication problem' OR 'communicative dysfunction*' OR 'agraphia*' OR 'alexia*' OR 'disfluency' OR 'articulat* disorder*' OR 'articulat* dysfunction' OR 'articulat* impairment' OR 'childhood onset fluency disorder*' OR 'developmental apraxia' OR 'fluency disorder' OR 'mis articulation' OR 'nonfluent speech' OR 'phonetic disorder' OR 'phonologic* impairment' OR 'phonological disorder*' OR 'speech disarticulation' OR 'speech nonfluency' OR 'speech sound disorder*' OR 'stammering' OR 'addh' OR 'attention deficit disorder*' OR 'overactive' OR 'hyperactiv*' OR 'adhd' OR 'add' OR 'attention deficit and disruptive behavio*r disorders' OR 'inattent*' OR 'minimal brain dysfunction' OR 'learning disabilit*' OR 'learning disorder*' OR 'learning disturbance*' OR 'developmental academic disabilit*' OR 'developmental academic disorder*' OR 'learning deficit' OR 'learning difficulty' OR 'learning impairment' OR 'learning problem' OR 'impaired learning' OR 'acalculia*' OR 'developmental disabilities of scholastic skills' OR 'developmental disorders of scholastic skills' OR 'primary dyscalculias' OR 'scholastic skills development disorders' OR 'word blindness*' OR 'handwriting disorder' OR 'math* disability' OR 'mathematics disorder' OR 'writing difficulty' OR 'strephosymbolia' OR 'secondary acalculia*' OR 'dyscalculia*' OR 'dysgraphia*' OR 'dyslectic child' OR 'dyslexia*' OR 'developmental dyspraxia' OR 'habit spasm' OR 'motor disability' OR 'motor disfunction' OR 'motor disorders' OR 'motor disturbance' OR 'motor dysfunction' OR 'motor impairment' OR 'motor skill* disorder*' OR 'developmental coordination disorder*' OR 'body rocking' OR 'stereotypic movement disorder*' OR 'tic disorder*' OR 'nervous tic' OR 'tourette* disease' OR 'tourette* disorder' OR 'tourette* syndrome' OR 'pervasive and specific developmental disorders' OR 'pervasive development* disorder*' OR 'disintegrative disorder' OR 'pdd' OR 'rett* disorder' OR 'rett* syndrome' OR 'morbus rett' OR 'rett disease' OR 'asperger* disease*' OR 'asperger* disorder*' OR 'asperger* syndrome' OR 'developmental speech or language disorders' OR 'reading problem' OR 'reading error' OR 'reading disturbance' OR 'reading disorder*' OR 'reading disabilit*' OR 'reading difficulty' OR 'developmental learning disorders' OR 'hyperkinet*' OR 'language disorder*' OR 'language deficiency' OR 'language impairment' OR 'language disability' OR 'central language imbalance' OR 'development* delay*' OR 'stuttering' OR 'opposition* defiant disorder*' OR 'conduct disorder*'
6	Domain for neurodevelopmental disorders	#4 OR #5
7	Emtree terms for Intervention population	'prepregnancy care'/exp OR 'pregnant person'/exp OR 'pregnancy'/exp OR 'maternal exposure'/exp OR 'pregnant woman'/exp OR 'mother'/exp
8	Search terms for Intervention population (title/abstract/keywords)	'childbearing' OR 'gestation' OR 'gravidit*' OR 'labo*r presentation' OR 'maternal exposure*' OR 'mother*' OR 'multigravidit*' OR 'nulligravidit*' OR 'preconception care' OR 'pregnanc*' OR 'pregnant people*' OR 'pregnant person' OR 'pregnant wom*n' OR 'prepregnancy care' OR 'primigravidit*' OR 'maternal' OR 'prenatal' OR 'antenatal' OR 'per-conception*' OR 'per-pregnancy' OR 'per-implantation' OR 'before pregnancy' OR 'before conception'
9	Domain for Intervention population	#7 OR #8
10	Emtree terms for meta-analyses and systematic review	'meta analysis'/exp OR 'systematic review'/exp
11	Search terms for meta-analyses and systematic review (title/abstract/keywords)	'meta analy*' OR 'systematic review' OR 'systematic review* as topic' OR 'meta regression' OR 'meta synthes*'
12	Domain for meta-analyses and systematic review	#10 OR #11
13	Total search string	#3 AND #6 AND #9 AND #12

Table S3. Search strategy for The Cochrane Library (14 records, accessed in 2025/06/30).

No.	Description or domain	Search terms
1	MeSH terms for folic acid (explode all trees)	Folic Acid

2	Search terms for folic acid (title/abstract/keywords)	'folate acid' OR 'folate' OR 'folate sodium' OR 'folavit' OR 'foldine' OR 'foliamin' OR 'folic*' OR 'folin*' OR 'folaci*' OR 'folart' OR 'folitab' OR 'folium acid' OR 'folivit' OR 'folsa*' OR 'folverlan' OR 'folvite' OR 'Vitamin B9' OR 'Vitamin M' OR 'acfol' OR 'acide folique ccd' OR 'acido folico' OR 'acifolic' OR 'apo folic' OR 'gravi fol' OR 'ingafol' OR 'lactobacillus casei' OR 'pteroylglutamic acid' OR '21179 34 0' OR '36653 55 1' OR '60672 17 5' OR '65165 91 5' OR '65165 92 6' OR '78168 16 8' OR '9p9w8ggu78'
3	Domain for folic acid	#1 OR #2
4	MeSH terms for neurodevelopmental disorders (explode all trees)	Neurodevelopmental Disorders OR Autism Spectrum Disorder OR Intellectual Disability OR Communication Disorders OR Language Disorders OR Speech Sound Disorder OR Childhood-Onset Fluency Disorder OR Social Communication Disorder OR Attention Deficit Disorder with Hyperactivity OR Specific Learning Disorder OR Dyslexia OR Agraphia OR Dyscalculia OR Motor Disorders OR Motor Skills Disorders OR Stereotypic Movement Disorder OR Tic Disorders OR Tourette Syndrome OR Child Development Disorders, Pervasive OR Rett Syndrome OR Asperger Syndrome OR Learning Disabilities
5	Search terms for neurodevelopmental disorders (title/abstract/keywords)	'neurodevelopmental disorder*' OR 'mental retardation' OR 'mental insufficiency' OR 'mental symptom' OR 'mental disorder*' OR 'mental abnormality' OR 'mental change' OR 'mental confusion' OR 'mental defect' OR 'mental deficienc*' OR 'mental disease' OR 'mental disturbance' OR 'mental health issue' OR 'mental* ill*' OR 'mental health problem' OR 'nervous twitch' OR 'neuropsychiatric disease*' OR 'neuropsychiatric disorder*' OR 'psychiatric illness' OR 'psychiatric symptom' OR 'psychi* disease*' OR 'psychi* disorder*' OR 'psychic disturbance' OR 'psychologic* disorder' OR 'psychologic* disturbance' OR 'autis*' OR 'ASD' OR 'clumsy child* syndrome' OR 'high functioning autism' OR 'kanner syndrome' OR 'intellectual disabilit*' OR 'intellectual disorder*' OR 'intellectual dysfunction' OR 'IQ' OR 'intellectual impairment' OR 'Idiocy' OR 'psychosocial mental retardation*' OR 'ID' OR 'IDD' OR 'communicatio* disorder*' OR 'communication disabilit*' OR 'communication disease' OR 'communication problem' OR 'communicative dysfunction*' OR 'agraphia*' OR 'alexia*' OR 'disfluency' OR 'articulat* disorder*' OR 'articulat* dysfunction' OR 'articulat* impairment' OR 'childhood onset fluency disorder*' OR 'developmental apraxia' OR 'fluency disorder' OR 'mis articulation' OR 'nonfluent speech' OR 'phonetic disorder' OR 'phonologic* impairment' OR 'phonological disorder*' OR 'speech disarticulation' OR 'speech nonfluency' OR 'speech sound disorder*' OR 'stammering' OR 'ADDH' OR 'attention deficit disorder*' OR 'overactive' OR 'hyperactiv*' OR 'ADHD' OR 'ADD' OR 'attention deficit and disruptive behavio*r disorders' OR 'inattent*' OR 'minimal brain dysfunction' OR 'learning disabilit*' OR 'learning disorder*' OR 'learning disturbance*' OR 'developmental academic disabilit*' OR 'developmental academic disorder*' OR 'learning deficit' OR 'learning difficulty' OR 'learning impairment' OR 'learning problem' OR 'impaired learning' OR 'acalculia*' OR 'developmental disabilities of scholastic skills' OR 'developmental disorders of scholastic skills' OR 'primary dyscalculias' OR 'scholastic skills development disorders' OR 'word blindness*' OR 'handwriting disorder' OR 'math* disability' OR 'mathematics disorder' OR 'writing difficulty' OR 'strephosymbolia' OR 'secondary acalculia*' OR 'dyscalculia*' OR 'dysgraphia*' OR 'dyslectic child' OR 'dyslexia*' OR 'developmental dyspraxia' OR 'habit spasm' OR 'motor disability' OR 'motor disfunction' OR 'motor disorders' OR 'motor disturbance' OR 'motor dysfunction' OR 'motor impairment' OR 'motor skill* disorder*' OR 'developmental coordination disorder*' OR 'body rocking' OR 'stereotypic movement disorder*' OR 'tic disorder*' OR 'nervous tic' OR 'tourette* disease' OR 'tourette* disorder' OR 'tourette* syndrome' OR 'pervasive and specific developmental disorders' OR 'pervasive development* disorder*' OR 'disintegrative disorder' OR 'PDD' OR 'rett* disorder' OR 'rett* syndrome' OR 'morbus rett' OR 'rett disease' OR 'asperger* disease*' OR 'asperger* disorder*' OR 'asperger* syndrome' OR 'developmental speech or language disorders' OR 'reading problem' OR 'reading error' OR 'reading disturbance' OR 'reading disorder*' OR 'reading disabilit*' OR 'reading difficulty' OR 'developmental learning disorders' OR 'hyperkinet*' OR 'language disorder*' OR 'language deficiency' OR 'language impairment' OR 'language disability' OR 'central language imbalance' OR 'development* delay*' OR 'stuttering' OR 'opposition* defiant disorder*' OR 'conduct disorder*'
6	Domain for neurodevelopmental disorders	#4 OR #5
7	MeSH terms for Intervention population (explode all trees)	Mothers OR Maternal Exposure OR Pregnant People OR Pregnancy OR Preconception Care OR Gravidity
8	Search terms for Intervention population (title/abstract/keywords)	'childbearing' OR 'gestation' OR 'gravidit*' OR 'labo*r presentation' OR 'maternal exposure*' OR 'mother*' OR 'multigravidit*' OR 'nulligravidit*' OR 'preconception care' OR 'pregnanc*' OR 'pregnant people*' OR 'pregnant person' OR 'pregnant wom*n' OR 'prepregnancy care' OR 'primigravidit*' OR 'maternal' OR 'prenatal' OR 'antenatal' OR 'per-conception*' OR 'per-pregnancy' OR 'per-implantation' OR 'before pregnancy' OR 'before conception'

9	Domain for Intervention population	#7 OR #8
10	MeSH terms for meta-analyses and systematic review (explode all trees)	Meta-Analysis as Topic OR Systematic Review
11	Search terms for meta-analyses and systematic review (title/abstract/keywords)	'meta analy*' OR 'Systematic Review' OR 'Systematic Review* as Topic' OR 'meta regression' OR 'meta synthes*'
12	Domain for meta-analyses and systematic review	#10 OR #11
13	Total search string	#3 AND #6 AND #9 AND #12

Table S4. Search strategy for Web of Science (106 records, accessed in 2025/06/30).

No.	Description or domain	Search terms
1	Search terms for folic acid (Topic)	Folic Acid OR folate acid OR folate OR folate sodium OR folavit OR foldine OR foliamin OR folic* OR folin* OR folaci* OR folart OR folitab OR folium acid OR folivit OR folsa* OR folverlan OR folvite OR Vitamin B9 OR Vitamin M OR acfol OR acide folique ccd OR acido folico OR acifolic OR apo folic OR gravi fol OR ingafol OR lactobacillus casei OR pteroylglutamic acid OR 21179 34 0 OR 36653 55 1 OR 60672 17 5 OR 65165 91 5 OR 65165 92 6 OR 78168 16 8 OR 9p9w8ggu78
2	Search terms for neurodevelopmental disorders (Topic)	Neurodevelopmental Disorders OR Autism Spectrum Disorder OR Intellectual Disability OR Communication Disorders OR Language Disorders OR Speech Sound Disorder OR Childhood-Onset Fluency Disorder OR Social Communication Disorder OR Attention Deficit Disorder with Hyperactivity OR Specific Learning Disorder OR Dyslexia OR Agraphia OR Dyscalculia OR Motor Disorders OR Motor Skills Disorders OR Stereotypic Movement Disorder OR Tic Disorders OR Tourette Syndrome OR Child Development Disorders, Pervasive OR Rett Syndrome OR Asperger Syndrome OR Learning Disabilities OR neurodevelopmental disorder* OR mental retardation OR mental insufficiency OR mental symptom OR mental disorder* OR mental abnormality OR mental change OR mental confusion OR mental defect OR mental deficienc* OR mental disease OR mental disturbance OR mental health issue OR mental* ill* OR mental health problem OR nervous twitch OR neuropsychiatric disease* OR neuropsychiatric disorder* OR psychiatric illness OR psychiatric symptom OR psychi* disease* OR psychi* disorder* OR psychic disturbance OR psychologic* disorder OR psychologic* disturbance OR autis* OR ASD OR clumsy child* syndrome OR high functioning autism OR kanner syndrome OR intellectual disabilit* OR intellectual disorder* OR intellectual dysfunction OR IQ OR intellectual impairment OR Idiocy OR psychosocial mental retardation* OR ID OR IDD OR communicatio* disorder* OR communication disabilit* OR communication disease OR communication problem OR communicative dysfunction* OR agraphia* OR alexia* OR disfluency OR articulat* disorder* OR articulat* dysfunction OR articulat* impairment OR childhood onset fluency disorder* OR developmental apraxia OR fluency disorder OR mis articulation OR nonfluent speech OR phonetic disorder OR phonologic* impairment OR phonological disorder* OR speech disarticulation OR speech nonfluency OR speech sound disorder* OR stammering OR attention deficit disorder* OR ADDH OR overactive OR hyperactiv* OR ADHD OR attention deficit and disruptive behavio*r disorders OR inattent* OR minimal brain dysfunction OR learning disabilit* OR learning disorder* OR learning disturbance* OR developmental academic disabilit* OR developmental academic disorder* OR learning deficit OR learning difficulty OR learning impairment OR learning problem OR impaired learning OR acalculia* OR developmental disabilities of scholastic skills OR developmental disorders of scholastic skills OR primary dyscalculias OR scholastic skills development disorders OR word blindness* OR handwriting disorder OR math* disability OR mathematics disorder OR writing difficulty OR strephosymbolia OR secondary acalculia* OR dyscalculia* OR dysgraphia* OR dyslectic child OR dyslexia* OR developmental dyspraxia OR habit spasm OR motor disability OR motor disfunction OR motor disorders OR motor disturbance OR motor dysfunction OR motor impairment OR motor skill* disorder* OR developmental coordination disorder* OR body rocking OR stereotypic movement disorder* OR tic disorder* OR nervous tic OR tourette* disease OR tourette* disorder OR tourette* syndrome OR specific developmental disorders OR pervasive development* disorder* OR disintegrative disorder OR PDD OR rett* disorder OR rett* syndrome OR morbus rett OR rett disease OR asperger* disease* OR asperger* disorder* OR asperger* syndrome OR developmental speech disorders OR developmental language

		disorders OR reading problem OR reading error OR reading disturbance OR reading disorder* OR reading disabilit* OR reading difficulty OR developmental learning disorders OR hyperkinet* OR language disorder* OR language deficiency OR language impairment OR language disability OR central language imbalance OR development* delay* OR stuttering OR opposition* defiant disorder* OR conduct disorder*
3	Search terms for Intervention population (Topic)	Mothers OR Maternal Exposure OR Pregnant People OR Pregnancy OR Preconception Care OR Gravidity OR childbearing OR gestation OR gravidit* OR labo*r presentation OR maternal exposure* OR mother* OR multigravidit* OR nulligravidit* OR preconception care OR pregnanc* OR pregnant people* OR pregnant person OR pregnant wom*n OR prepregnancy care OR primigravidit* OR maternal OR prenatal OR antenatal OR per-conception* OR per-pregnancy OR per-implantation OR before pregnancy OR before conception
4	Search terms for meta-analyses and systematic review (Topic)	Meta-Analysis as Topic OR Systematic Review OR meta analy* OR Systematic Review OR Systematic Review* as Topic OR meta regression OR meta synthes*
5	Total search string	#1 AND #2 AND #3 AND #4

Table S5. Search strategy for Medline (Ovid) (42 records, accessed in 2025/06/30).

No.	Description or domain	Search terms
1	MeSH terms for folic acid	Folic Acid/
2	Search terms for folic acid (title/abstract/keywords)	folate acid or folate or folate sodium or folavit or foldine or foliamin or folic* or folin* or folaci* or folart or folitab or folium acid or folivit or folsa* or folverlan or folvite or Vitamin B9 or Vitamin M or acfol or acide folique ccd or acido folico or acifolic or apo folic or gravi fol or ingafol or lactobacillus casei or pteroylglutamic acid or "21179 34 0" or 36653 55 1 or 60672 17 5 or 65165 91 5 or 65165 92 6 or 78168 16 8 or 9p9w8ggu78
3	Domain for folic acid	#1 or #2
4	MeSH terms for neurodevelopmental disorders	Neurodevelopmental Disorders/ or Autism Spectrum Disorder/ or Intellectual Disability/ or Communication Disorders/ or Language Disorders/ or Speech Sound Disorder/ or Childhood-Onset Fluency Disorder/ or Social Communication Disorder/ or Attention Deficit Disorder with Hyperactivity/ or Specific Learning Disorder/ or Dyslexia/ or Agraphia/ or Dyscalculia/ or Motor Disorders/ or Motor Skills Disorders/ or Stereotypic Movement Disorder/ or Tic Disorders/ or Tourette Syndrome/ or Child Development Disorders, Pervasive/ or Rett Syndrome/ or Asperger Syndrome/ or Learning Disabilities/
5	Search terms for neurodevelopmental disorders (title/abstract/keywords)	neurodevelopmental disorder* or mental retardation or mental insufficiency or mental symptom or mental disorder* or mental abnormality or mental change or mental confusion or mental defect or mental deficienc* or mental disease or mental disturbance or mental health issue or mental* ill* or mental health problem or nervous twitch or neuropsychiatric disease* or neuropsychiatric disorder* or psychiatric illness or psychiatric symptom or psychi* disease* or psychi* disorder* or psychic disturbance or psychologic* disorder or psychologic* disturbance or autis* or ASD or clumsy child* syndrome or high functioning autism or kanner syndrome or intellectual disabilit* or intellectual disorder* or intellectual dysfunction or IQ or intellectual impairment or Idiocy or psychosocial mental retardation* or ID or IDD or communicatio* disorder* or communication disabilit* or communication disease or communication problem or communicative dysfunction* or agraphia* or alexia* or disfluency or articulat* disorder* or articulat* dysfunction or articulat* impairment or childhood onset fluency disorder* or developmental apraxia or fluency disorder or mis articulation or nonfluent speech or phonetic disorder or phonologic* impairment or phonological disorder* or speech disarticulation or speech nonfluency or speech sound disorder* or stammering or attention deficit disorder* or ADDH or overactive or hyperactiv* or ADHD or disruptive behavio*r disorders or inattent* or minimal brain dysfunction or learning disabilit* or learning disorder* or learning disturbance* or developmental academic disabilit* or developmental academic disorder* or learning deficit or learning difficulty or learning impairment or learning problem or impaired learning or acalculia* or developmental disabilities of scholastic skills or developmental disorders of scholastic skills or primary dyscalculias or scholastic skills development disorders or word blindness* or handwriting disorder or math* disability or mathematics disorder or writing difficulty or strephosymbolia or secondary acalculia* or dyscalculia* or dysgraphia* or dyslectic child or dyslexia* or developmental dyspraxia or habit spasm or motor disability or motor disfunction or motor disorders or motor disturbance or motor dysfunction or motor impairment or motor skill* disorder* or developmental coordination disorder* or body rocking or stereotypic movement disorder* or tic disorder* or nervous tic or tourette* disease or tourette* disorder or tourette* syndrome or specific developmental disorders or pervasive development* disorder* or disintegrative disorder or PDD or rett* disorder or rett* syndrome or morbus rett or rett disease or asperger* disease* or asperger* disorder* or asperger* syndrome or developmental speech disorders or developmental language disorders or reading problem or reading error or reading disturbance or reading disorder* or reading disabilit* or reading difficulty or developmental learning disorders or hyperkinet* or

		language disorder* or language deficiency or language impairment or language disability or central language imbalance or development* delay* or stuttering or opposition* defiant disorder* or conduct disorder*
6	Domain for neurodevelopmental disorders	#4 or #5
7	MeSH terms for Intervention population	Mothers/ or Maternal Exposure/ or Pregnant People/ or Pregnancy/ or Preconception Care/ or Gravidity/
8	Search terms for Intervention population (title/abstract/keywords)	childbearing or gestation or gravidit* or labo*r presentation or maternal exposure* or mother* or multigravidit* or nulligravidit* or preconception care or pregnanc* or pregnant people* or pregnant person or pregnant wom*n or prepregnancy care or primigravidit* or maternal or prenatal or antenatal or per-conception* or per-pregnancy or per-implantation or before pregnancy or before conception
9	Domain for Intervention population	#7 or #8
10	MeSH terms for meta-analyses and systematic review	Systematic Review/ or Meta-Analysis as Topic/
11	Search terms for meta-analyses and systematic review (title/abstract/keywords)	meta analy* or Systematic Review or Systematic Review* as Topic or meta regression or meta synthes*
12	Domain for meta-analyses and systematic review	#10 or #11
13	Total search string	#3 and #6 and #9 and #12

Table S6. AMSTAR-2 scores for outcomes of neurodevelopmental disorders in meta-analyses/systematic reviews.

Author（year）	Study design	1	2	3	4	5	6	7	8	9	10	11	12	13	14	15	16	Rating
Wang et al [1]	Nonrandomized studies	Yes	No	Yes	PY	Yes	Yes	PY	PY	No	No	Yes	No	No	No	Yes	Yes	CL
Guo et al [2]	Nonrandomized studies	Yes	PY	Yes	Yes	Yes	Yes	Yes	PY	Yes	No	Yes	Yes	Yes	Yes	Yes	Yes	High
Yu et al [3]	Nonrandomized studies	Yes	No	Yes	PY	Yes	Yes	PY	PY	PY	No	Yes	No	No	Yes	Yes	Yes	CL
Liu, et al [4]	Nonrandomized studies	Yes	PY	Yes	PY	Yes	Yes	PY	Yes	Yes	No	Yes	Yes	Yes	Yes	Yes	Yes	High
Jia et al [5]	Nonrandomized studies	Yes	No	Yes	PY	Yes	Yes	PY	Yes	Yes	No	Yes	No	No	Yes	No	Yes	CL
Vazque et al [6]	Nonrandomized studies	Yes	No	Yes	PY	Yes	Yes	PY	Yes	Yes	No	Yes	Yes	Yes	Yes	Yes	Yes	Low
Friel et al [7]	Nonrandomized studies	Yes	Yes	Yes	PY	Yes	Yes	PY	Yes	Yes	No	Yes	Yes	Yes	Yes	Yes	Yes	High
Li et al [8]	Nonrandomized studies	Yes	No	Yes	PY	No	No	No	Yes	PY	Yes	Yes	Yes	Yes	Yes	No	Yes	CL
Chen et al [9]	Nonrandomized studies	Yes	Yes	Yes	Yes	Yes	Yes	PY	Yes	Yes	No	Yes	Yes	Yes	Yes	Yes	Yes	High
Sampaio et al [10]	Nonrandomized studies	PY	No	No	PY	No	No	PY	PY	No	No	No meta-analysis	No meta-analysis	No	No	No meta-analysis	Yes	CL

Hoxha et al [11]	Nonrandomized studies	PY	PY	No	PY	Yes	Yes	PY	PY	No	No	No meta-analysis	No meta-analysis	Yes	Yes	No meta-analysis	Yes	Low
Gao et al [12]	Nonrandomized studies	Yes	Yes	Yes	PY	Yes	Yes	Yes	Yes	PY	No	No meta-analysis	No meta-analysis	Yes	Yes	No meta-analysis	Yes	Mod-erate
DeVilbiss et al [13]	Nonrandomized studies	PY	No	Yes	No	Yes	No	PY	PY	No	No	No meta-analysis	No meta-analysis	No	Yes	No meta-analysis	Yes	CL
Zhong et al [14]	Nonrandomized studies	Yes	No	Yes	PY	Yes	Yes	PY	Yes	No	No	No meta-analysis	No meta-analysis	Yes	Yes	No meta-analysis	Yes	Low
Viswanathan et al [15]	Nonrandomized studies	Yes	Yes	Yes	Yes	Yes	Yes	PY	PY	Yes	No	No meta-analysis	No meta-analysis	Yes	Yes	No meta-analysis	Yes	High
Vasconcelos et al [16]	Nonrandomized studies	Yes	Yes	Yes	Yes	Yes	Yes	PY	Yes	Yes	No	No meta-analysis	No meta-analysis	Yes	Yes	No meta-analysis	Yes	High
Cheng et al [17]	Nonrandomized studies	PY	No	Yes	No	No	No	No	PY	No	No	No meta-analysis	No meta-analysis	No	No	No meta-analysis	Yes	CL
Chmielewska et al [18]	Nonrandomized studies	Yes	No	Yes	PY	No	No	No	PY	PY	No	No meta-analysis	No meta-analysis	Yes	No	No meta-analysis	Yes	CL
Sargoor et al [19]	Nonrandomized studies	Yes	PY	Yes	PY	Yes	Yes	PY	PY	PY	No	No meta-analysis	No meta-analysis	Yes	Yes	No meta-analysis	Yes	High
Chimeh et al [20]	Nonrandomized studies	PY	No	No	PY	Yes	No	PY	Yes	No	No	No meta-analysis	No meta-analysis	Yes	Yes	No meta-analysis	Yes	CL
Freedman et al [21]	Nonrandomized studies	Yes	No	Yes	PY	No	No	No	PY	No	No	No meta-analys	No meta-analys	Yes	Yes	No meta-analys	Yes	CL
Chamova et al [22]	Nonrandomized studies	PY	No	Yes	PY	Yes	Yes	PY	Yes	No	No	No meta-analys	No meta-analys	No	Yes	No meta-analys	Yes	CL

Table S7. GRADE ratings of meta-analyses/systematic reviews of neurodevelopmental disorders.

Author（year)	No of studies	Study design	Risk of Bias	Inconsistency	Indirectness	Imprecision	Publication bias	Large effect	Plausible Confounding	Dose-re-sponse	Quality
					ASD						
Wang et al [1]	10	observational studies	Serious	Serious	No serious indirectness	No serious imprecision	Undetected	No	Would not re-duce effect	No	⊕OOO VERY LOW
Guo et al [2]	8	observational studies	Serious	Serious	No serious indirectness	Serious	Undetected	No	Would not re-duce effect	No	⊕OOO VERY LOW
Yu et al [3]	10	observational studies	No serious indirectness	Serious	No serious indirectness	No serious indirectness	Undetected	No	Would not re-duce effect	No	⊕⊕⊕O MODERATE
Liu, et al [4]	10	observational studies	Serious	Serious	No serious indirectness	No serious indirectness	Undetected	Large	Would reduce effect	Yes	⊕⊕⊕O MODERATE

Jia et al [5]	17	observational studies	Serious	Serious	No serious indirectness	No serious indirectness	Strongly suspected	No	Would not reduce effect	No	⊕OOO VERY LOW
Vazque et al [6]	9	observational studies	No serious indirectness	Serious	No serious indirectness	No serious indirectness	Strongly suspected	Large	Would not reduce effect	Yes	⊕⊕OO LOW
Friel et al [7]	10	observational studies	Serious	Serious	No serious indirectness	Serious	Undetected	No	Would not reduce effect	No	⊕OOO VERY LOW
Li et al [8]	6	observational studies	Serious	Serious	No serious indirectness	No serious indirectness	Undetected	No	Would not reduce effect	Yes	⊕OOO VERY LOW
Chen et al [9]	16	observational studies	Serious	Serious	No serious indirectness	No serious indirectness	Strongly suspected	No	Would not reduce effect	Yes	⊕OOO VERY LOW
Sampaio et al [10]	17	observational studies	Serious	No serious indirectness	No serious indirectness	Serious	Undetected	No	Would not reduce effect	Yes	⊕OOO VERY LOW
Hoxha et al [11]	10	observational studies	Serious	Serious	No serious indirectness	No serious indirectness	Undetected	No	Would not reduce effect	No	⊕OOO VERY LOW
Gao et al [12]	4	observational studies	Serious	No serious indirectness	No serious indirectness	Serious	Undetected	No	Would not reduce effect	No	⊕OOO VERY LOW
DeVilbiss et al [13]	10	observational studies	Serious	No serious indirectness	No serious indirectness	No serious indirectness	Undetected	No	Would not reduce effect	Yes	⊕⊕OO LOW
Zhong et al [14]	15	observational studies	Serious	Serious	No serious indirectness	No serious indirectness	Undetected	No	Would not reduce effect	No	⊕OOO VERY LOW
Viswanathan et al [15]	7	observational studies	Serious	No serious indirectness	No serious indirectness	No serious indirectness	Undetected	No	Would not reduce effect	No	⊕OOO VERY LOW
Vasconcelos et al [16]	14	observational studies	Serious	Serious	No serious indirectness	No serious indirectness	Undetected	No	Would not reduce effect	No	⊕OOO VERY LOW
Cheng et al [17]	3	observational studies	Serious	No serious indirectness	No serious indirectness	No serious indirectness	Undetected	No	Would not reduce effect	Yes	⊕⊕OO LOW
Chmielewska et al [18]	2	observational studies	Serious	No serious indirectness	No serious indirectness	No serious indirectness	Undetected	No	Would not reduce effect	No	⊕OOO VERY LOW

ADHD										
Li et al [8]	5	observational studies	Serious	Serious	No serious indirectness	No serious indirectness	Undetected	No	Would not reduce effect	No	⊕OOO VERY LOW
Cheng et al [17]	6	observational studies	Serious	No serious indirectness	No serious indirectness	No serious indirectness	Undetected	No	Would not reduce effect	No	⊕OOO VERY LOW
Gao et al [12]	1	observational studies	Serious	No serious indirectness	No serious indirectness	Serious	Undetected	No	Would not reduce effect	No	⊕OOO VERY LOW
Chmielewska et al [18]	1	observational studies	Serious	No serious indirectness	No serious indirectness	No serious indirectness	Undetected	No	Would not reduce effect	No	⊕OOO VERY LOW
Sargoor et al [19]	2	observational studies	Serious	No serious indirectness	No serious indirectness	No serious indirectness	Undetected	No	Would not reduce effect	No	⊕OOO VERY LOW
Motor Development										
Vazque et al [6]	4	observational studies	Serious	No serious indirectness	No serious indirectness	Serious	Undetected	No	Would not reduce effect	No	⊕OOO VERY LOW
Chen et al [9]	4	observational studies	Serious	Serious	No serious indirectness	Serious	Strongly suspected	No	Would not reduce effect	No	⊕OOO VERY LOW
Gao et al [12]	3	observational studies	Serious	Serious	No serious indirectness	No serious indirectness	Undetected	No	Would not reduce effect	Yes	⊕OOO VERY LOW
Chmielewska et al [18]	1	observational studies	Serious	No serious indirectness	No serious indirectness	No serious indirectness	Undetected	No	Would not reduce effect	No	⊕OOO VERY LOW
Sargoor et al [19]	4	observational studies	Serious	Serious	No serious indirectness	No serious indirectness	Undetected	Large	Would not reduce effect	Yes	⊕⊕OO LOW
Intellectual or cognitive development										
Chen et al [9]	5	observational studies	Serious	Serious	No serious indirectness	Serious	Strongly suspected	No	Would not reduce effect	No	⊕OOO VERY LOW
Gao et al [12]	3	observational studies	Serious	No serious indirectness	No serious indirectness	Serious	Undetected	No	Would not reduce effect	No	⊕OOO VERY LOW

Sargoor et al [19]	2	observational studies	Serious	No serious in-directness	No serious indirectness	No serious indirectness	Undetected	Large	Would not re-duce effect	Yes	⊕⊕⊕O MODERATE
Chimeh et al[20]	1	observational studies	Serious	No serious in-directness	No serious indirectness	No serious indirectness	Undetected	No	Would not re-duce effect	No	⊕OOO VERY LOW
					Behavior development						
Chen et al [9]	3	observational studies	Serious	No serious in-directness	No serious indirectness	No serious indirectness	Undetected	No	Would not re-duce effect	No	⊕OOO VERY LOW
Gao et al [12]	2	observational studies	Serious	No serious in-directness	No serious indirectness	No serious indirectness	Undetected	No	Would not re-duce effect	No	⊕OOO VERY LOW
Chmielewska et al [18]	1	observational studies	Serious	No serious in-directness	No serious indirectness	No serious indirectness	Undetected	No	Would not re-duce effect	No	⊕OOO VERY LOW
Freedman et al [21]	2	observational studies	Serious	No serious in-directness	No serious indirectness	No serious indirectness	Undetected	No	Would not re-duce effect	No	⊕OOO VERY LOW
					Language development						
Chen et al [9]	4	observational studies	Serious	Serious	No serious indirectness	Serious	Undetected	No	Would not re-duce effect	No	⊕OOO VERY LOW
Gao et al [12]	1	observational studies	Serious	No serious in-directness	No serious indirectness	No serious indirectness	Undetected	No	Would not re-duce effect	Yes	⊕⊕OO LOW
Chmielewska et al [18]	3	observational studies	Serious	No serious in-directness	No serious indirectness	No serious indirectness	Undetected	No	Would not re-duce effect	No	⊕OOO VERY LOW
Sargoor et al [19]	4	observational studies	Serious	No serious in-directness	No serious indirectness	No serious indirectness	Undetected	Large	Would not re-duce effect	Yes	⊕⊕⊕O MODERATE
					Mental development						
Vazque et al [6]	5	observational studies	Serious	No serious in-directness	No serious indirectness	Serious	Strongly suspected	No	Would not re-duce effect	No	⊕OOO VERY LOW
Gao et al [12]	1	observational studies	Serious	No serious in-directness	No serious indirectness	No serious indirectness	Undetected	No	Would not re-duce effect	Yes	⊕⊕OO LOW

Sargoor et al [19]	4	observational studies	Serious	No serious indirectness	No serious indirectness	No serious indirectness	Undetected	No	Would not reduce effect	No	⊕OOO<br>VERY LOW
					Neurodevelopment						
Chamova et al [22]	1	observational studies	Serious	No serious indirectness	No serious indirectness	No serious indirectness	Undetected	No	Would not reduce effect	No	⊕OOO<br>VERY LOW

Table S8. Results of heterogeneity (I^2 and Q test) and publication bias (Egger test, over-significance test).

**Author（year)**	**Number of studies included (k)**	**Recalculated summary effect (95% CI)**	**I^2 (%)**	**Q test p-value**	**Egger's test p-value**	**excess significance test (O/E, p-value)**	**Note**
				ASD			
Wang et al [1]	16	RR = 0.768 (0.638-0.925)	60.20	0.001	0.005	1.19, $p<0.001$	No stratification was made by research type.
Guo et al [2]	8	OR = 0.909 (0.730-1.133)<br>HR = 0.661 (0.374-1.168)	OR: 76.28<br>HR: 97.30	OR: <0.001<br>HR: <0.001	NA	NA	$p≥0.05$, no bias was tested.
Yu et al [3]	10	OR = 0.798 (0.669-0.952)	60.34	0.001	0.011	1.20, $p<0.001$	
Liu, et al [4]	10	NA	NA	NA	NA	NA	The description in the result section does not match the forest map.
Jia et al [5]	17	NA	NA	NA	NA	NA	Information on folic acid supplementation for mothers in the case group/control group could not be obtained. It is also impossible to obtain the case information of ASD in the exposed group/non-exposed group offspring, and the effect value of each original study cannot be obtained.
Vazque et al [6]	9	OR = 0.585 (0.457-0.750)	87.28	<0.001	0.145	21.95, $p<0.001$	
Friel et al [7]	10	RR = 0.736 (0.571-0.949)	94.31	<0.001	0.441	6.10, $p<0.001$	
Li et al [8]	6	RR = 0.642 (0.456-0.902)<br>Folic acid only: RR = 0.519 (0.261-1.031)	96.08<br>Folic acid only: 97.09	<0.001<br>Folic acid only: <0.001	0.322<br>Folic acid only: NA	5.23, $p<0.001$<br>Folic acid only: NA	When folic acid was supplemented alone, $p≥0.05$, and bias was not tested.
Chen et al [9]	16	Case-control studies: OR = 0.673 (0.431-1.051)<br>Cohort studies: OR = 0.690 (0.508-0.938)	Case-control studies: 86.42<br>Cohort studies: 91.54	Case-control studies: <0.001<br>Cohort studies: <0.001	Case-control studies: NA<br>Cohort studies: 0.383	Case-control studies: NA<br>Cohort studies: 2.533, $p<0.001$	Case control group: $p≥0.05$, no bias was tested
				ADHD			

Chen et al [9]	6	OR = 0.864 (0.784-0.952)	0	0.678	0.741	0.579, *p* = 0.265	
Motor Development							
Chen et al [9]	4	Beta = 1.019 (-0.886-2.924)	60.43	0.056	NA	NA	*p*≥0.05, no bias was tested.
Vazque et al [6]	4	SMD = -0.020 (-0.080-0.039)	20.77	0.179	NA	NA	*p*≥0.05, no bias was tested.
Intellectual or cognitive development							
Chen et al [9]	5	Beta = 1.297 (-1.613-4.206)	72.64	0.006	NA	NA	*p*≥0.05, no bias was tested.
Behavioral and emotional problems							
Chen et al [9]	3	OR = 0.752 (0.625-0.906)	0	0.520	0.026	NA	If the number of studies is less than 4, the excess probability test cannot be conducted
Language development							
Chen et al [9]	4	Beta = 0.776 (-1.166-2.719)	63.09	0.043	NA	NA	*p*≥0.05, no bias was tested.
Mental Development							
Vazque et al [6]	7	SMD = -0.057 (-0.116-0.002) Non-randomised studies: SMD = -0.048 (-0.109-0.014)	35.55 Non-randomised studies: 26.42	0.036 Non-randomised studies: 0.112	NA	NA	*p*≥0.05, no bias was tested.

Heterogeneity judgment criteria: If $I^2 \geq 50\%$ and Q test $p<0.05$, it is considered significant heterogeneity; Egger's test: Calculated only when the pooled effect size $p<0.05$ and $k \geq 3$; Excessive significance test: Calculated only when the pooled effect size $p<0.05$ and $k \geq 4$; The random effects model was used for all analyses.

Table S9. ASD original study information extraction table.

Study	Study design	Dose	Supplementary period	Country	Total participants	Cases	Effect sizes and 95% confidence intervals	Source Meta
Chen, Q. 2014 [23]	cross-sectional study	NA	During pregnancy	China	4754	14	OR = 0.18 (0.05-0.62)	Chen et al [9]
Li, X. 2015 [24]	case-control study	NA	Periconceptional period	China	362	181	OR = 0.07 (0.01-0.45)	Chen et al [9]
Su, Y. Y. 2012 [25]	case-control study	NA	During pregnancy	China	297	96	OR = 0.92 (0.40-2.08)	Wang et al [1]
Zhang, X. 2015 [1]	case-control study	NA	During pregnancy	China	733	193	OR = 0.40 (0.17-0.94)	Wang et al [1]
Jiang, H. 2016 [26]	cross-sectional study	NA	During pregnancy	China	8842	767	OR = 0.31 (0.10-0.94)	Chen et al [9]
Nilsen, R. M. 2013a [27]	case-control study	NA	During pregnancy	Norway	89836	234	OR = 0.85 (0.65-1.11)	Chen et al [9]
Nilsen, R. M. 2013b [27]	cohort study	NA	During pregnancy	Norway	507856	2072	OR = 0.86 (0.78-0.95)	Chen et al [9]
Sure'n, P. 2013 (AD) [28]	cohort study	NA	Periconceptional period	Norway	85176	114	OR = 0.51 (0.35-0.73)	Liu, et al [4]
Sure'n, P. 2013 (AS) [28]	cohort study	NA	Periconceptional period	Norway	30117	48	OR = 0.58 (0.33-1.03)	Liu, et al [4]

Sure'n, P. 2013 (PPD-NOS) [28]	cohort study	NA	Periconceptional period	Norway	29192	91	OR = 0.87 (0.57-1.34)	Liu, et al [4]
Schmidt, R. J. 2012 [29]	case-control study	≥600μg/d	Early pregnancy	The United States	707	429	OR = 0.61 (0.41-0.90)	Chen et al [9]
Virk, J. 2016 [30]	cohort study	NA	Early pregnancy	Denmark	19042	300	OR = 0.93 (0.73-1.17)	Liu, et al [4]
Sun, Y. L. 2016 [31]	cohort study	NA	During pregnancy	China	3663	290	OR = 0.95 (0.73-1.24)	Chen et al [9]
DeSoto, M. C. 2012 [32]	case-control study	NA	During pregnancy	The United States	1008	256	OR = 2.34 (1.14-4.81)	Chen et al [9]
DeVilbiss, A. 2017 [33]	cohort study	NA	Early pregnancy	Sweden	94684	4412	OR = 1.29 (0.99-1.67)	Chen et al [9]
Levine, S. Z. 2018 [34]	cohort study	NA	During pregnancy	Israel	45300	572	OR = 0.32 (0.26-0.41)	Chen et al [9]
Strøm, M. 2018 [35]	cohort study	NA	Mid-pregnancy	Denmark	87210	1234	OR = 0.99 (0.81-1.21)	Chen et al [9]
Schmidt, R. J. 2019 [36]	cohort study	≥600μg/d	Early pregnancy	The United States	241	55	OR = 0.51 (0.31-0.82)	Chen et al [9]
Schmidt, R. J. 2017 (600μg) [37]	case-control study	600μg/d	Early pregnancy	The United States	676	394	OR = 0.68 (0.50-0.93)	Liu, et al [4]
Schmidt, R. J. 2017 （800μg） [37]	case-control study	800μg/d	Early pregnancy	The United States	676	394	OR = 0.66 (0.48-0.90)	Liu, et al [4]
Li, Y. M. 2018 (During pregnancy) [38]	case-control study	NA	During pregnancy	China	675	344	OR = 0.64 (0.41-1.00)	Liu, et al [4]
Li, Y. M. 2018 (Before pregnancy）[38]	case-control study	NA	Before pregnancy	China	656	322	OR = 0.95 (0.61-1.50)	Liu, et al [4]
Tan, M. 2019 [39]	case-control study	NA	Periconceptional period	China	617	416	OR = 0.52 (0.35-0.78)	Chen et al [9]
Moser, S. S. 2019 [40]	case-control study （Nested Case-Control Study）	> 1000μg/day	During pregnancy	The United States	21895	2009	OR = 1.10 (0.98-1.24)	Chen et al [9]
Braun, J. M. 2014 (4 years) [41]	cohort study	NA	Mid-pregnancy	The United States	178	21	OR = 0.17 (0.06-0.48)	Vazque et al [6]
Braun, J. M. 2014 (5 years) [41]	cohort study	NA	Mid-pregnancy	The United States	193	34	OR = 0.14 (0.06-0.33)	Vazque et al [6]
Goodrich, A. J. 2018 [42]	case-control study	＞800μg/d	Early pregnancy	The United States	606	346	OR = 0.67 (0.48-0.92)	Vazque et al [6]
Schmidt, R. J. 2011 [43]	case-control study	NA	Periconceptional period	The United States	483	254	OR = 0.57 (0.39-0.83)	Vazque et al [6]
Al-maqati, T. N. 2021 [44]	case-control study	NA	Early pregnancy	Saudi Arabia	305	160	OR = 0.34 (0.17-0.68)	Chen et al [9]

Table S10. Subgroup analysis of original research on autism spectrum disorders (ASD).

Subgroup variable	Category	Research numbers	OR（95% CI）	I^2 (%)	Results of pairwise comparisons
**Research design**	Case-control study	13	0.69 (0.54, 0.88)	80.2	Vs Cohort: p=0.7636;<br>Vs Cross-sectional: p=0.0148*
	Cohort study	9	0.65 (0.48, 0.88)	93.3	Vs Case-control: *p*=0.7636;<br>Vs Cross-sectional: *p*=0.0245*
	Cross-sectional study	2	0.24 (0.11, 0.56)	0.0	Vs Cohort: *p*=0.0245*;<br>Vs Case-control: *p*=0.0148*
**Sample size**	Small (≤651)	8	0.47 (0.34, 0.65)	63.5	Vs Medium: *p*=0.1240;<br>Vs Large: *p*=0.0119*
	Medium (652-10678)	8	0.68 (0.48, 0.95)	70.8	Vs Small: *p*=0.1240;<br>Vs Large: *p*=0.4570
	Large (>10678)	8	0.80 (0.62, 1.04)	93.5	Vs Small: *p*=0.0119*;<br>Vs Medium: *p*=0.4570
**Dose**	NA	19	0.63 (0.50, 0.79)	87.7	*p*=0.5608
	>400μg/d	5	0.71 (0.51, 0.99)	84.6	
**Supplementary period**	Periconceptional period	4	0.51 (0.38, 0.68)	33.4	Vs Early pregnancy: *p*=0.1066;<br>Vs During pregnancy: *p*=0.1479
	Early pregnancy	7	0.71 (0.54, 0.94)	78.2	Vs Periconceptional period: *p*=0.1066;<br>Vs During pregnancy: *p*=0.8235
	During pregnancy	13	0.68 (0.53, 0.89)	90.8	Vs Periconceptional period: *p*=0.1479;<br>Vs Early pregnancy: *p*=0.8235
**Region**	East Asia	8	0.52 (0.35, 0.78)	67.5	Vs Nordic Europe: *p*=0.0132*;<br>Vs North America: *p*=0.3516;<br>Vs Middle East: *p*=0. 0365*
	Nordic Europe	6	0.90 (0.76, 1.06)	72.7	Vs East Asia: *p*=0.0132*;<br>Vs North America: *p*=0.1372;<br>Vs Middle East: *p*<0.001*
	North America	8	0.67 (0.47, 0.95)	87.8	Vs East Asia: *p*=0.3516;<br>Vs Nordic Europe: *p*=0.1372;<br>Vs Middle East: *p*<0.001*
	Middle East	2	0.32 (0.26, 0.40)	0.0	Vs East Asia: *p*=0.0365*;

					Vs Nordic Europe: p<0.001*; Vs North America: p<0.001*
**Mandatory folic acid supplementation policy**	Yes	8	0.67 (0.47, 0.95)	87.8	p=0.8312
	No	16	0.64 (0.51, 0.81)	87.4	
**AMSTAR-2 rating of source meta**	High	19	0.70 (0.58, 0.85)	88.5	p=0.1615
	Low or Critically Low	5	0.49 (0.31, 0.78)	70.0	

Table S11. Motor Development Original Study Information Extraction Table.

Study	Study design	Dose	Supplementary period	Country	Total participants	Effect sizes and 95% confidence intervals	Source Meta
Julvez, J. 2009 [45]	Cohort Study	NA	During pregnancy	Spain	420	β=4.55 (1.28, 7.81)	Chen et al [9]
Del Río García, C. 2009 [46]	Cohort Study	<400μg/d	Early pregnancy	Mexico	253	β=-0.40 (-2.1, 1.4)	Chen et al [9]
Chatzi, L. 2012 [47]	Cohort Study	＞1000~5000μg/d	Early pregnancy	Greece	419	SMD=0.22 (-0.09, 0.53)	Vazque et al [6]
		>5000μg/d			180	SMD=0.17 (-0.16, 0.51)	
Valera-Gran, D. 2014 [48]	Cohort Study	1000–5000μg/d	Before and during pregnancy	Spain	2213	β=-0.02 (-1.89, 1.86)	Chen et al [9]
Polanska, K. 2015 [49]	Cohort Study	NA	Pre-pregnancy or early pregnancy	Poland	538	β=1.50 (-1.70, 4.80)	Chen et al [9]
Valera-Gran, D. 2017 (Asturias) [50]	Cohort Study	400-1000μg/day	Periconception or mid-to-late pregnancy	Spain	209	SMD=0.16 (-0.12, 0.43)	Vazque et al [6]
		>1000-5000μg/day			246	SMD=-0.17 (-0.42, 0.09)	
Valera-Gran, D. 2017 (Guipuzkoa) [50]	Cohort Study	400-1000μg/day	Periconception or mid-to-late pregnancy	Spain	251	SMD=-0.39 (-0.74, -0.03)	Vazque et al [6]
		>1000-5000μg/day			326	SMD=0.00 (-0.23, 0.23)	
Valera-Gran, D. 2017 (Sabadell) [50]	Cohort Study	400-1000μg/day	Periconception or mid-to-late pregnancy	Spain	390	SMD=0.05 (-0.24, 0.34)	Vazque et al [6]
		>1000-5000μg/day			406	SMD=-0.12 (-0.38, 0.14)	
Valera-Gran, D. 2017 (Valencia) [50]	Cohort Study	400-1000μg/day	Periconception or mid-to-late pregnancy	Spain	392	SMD=-0.09 (-0.30, 0.12)	Vazque et al [6]
		>1000-5000μg/day			396	SMD=0.04 (-0.17, 0.25)	

Table S12. Mental development original study information extraction table.

Study	Study design	Dose	Supplementary period	Country	Total participants	Effect sizes and 95% confidence intervals	Source Meta
Campoy, C. 2011 [51]	cohort study	400µg/day	Mid-pregnancy	Germany, Spain, Hungary	80	SMD=-0.15 (-0.59, 0.30)	Vazque et al [6]
Czeizel, A. E. 1994 [52]	randomised controlled trial	800µg/day	Periconceptional period	Hungary	2082	SMD=0.00 (-0.09, 0.09)	
Dobo, M. 1998 [53]	randomised controlled trial	800µg/day	Periconceptional period	Hungary	336	SMD=-0.33 (-0.54, -0.11)	
Polańska, K. 2015 (12 months) [49]	cohort study	400µg/day	Pre-pregnancy or early pregnancy	Poland	500	SMD=0.03 (-0.15, 0.21)	
Polańska, K. 2015 (24 months) [49]	cohort study	400µg/day	Pre-pregnancy or early pregnancy	Poland	340	SMD=0.01 (-0.21, 0.22)	
Valera-Gran, D. 2014 (Asturias) [48]	cohort study	400-1000µg/day	Periconception or mid-to-late pregnancy	Spain	254	SMD=-0.11 (-0.36, 0.14)	
		>1000-5000µg/day			274	SMD=-0.24 (-0.47, 0.00)	
		>5000µg/d			174	SMD=-0.04 (-0.44, 0.36)	
Valera-Gran, D. 2014 (Guipuzkoa) [48]	cohort study	400-1000µg/day	Periconception or mid-to-late pregnancy	Spain	385	SMD=-0.06 (-0.33, 0.21)	
		>1000-5000µg/day			470	SMD=0.19 (-0.01, 0.38)	
		>5000µg/d			337	SMD=0.31 (-0.20, 0.83)	
Valera-Gran, D. 2014 (Sabadell) [48]	cohort study	400-1000µg/day	Periconception or mid-to-late pregnancy	Spain	474	SMD=-0.44 (-0.70, -0.17)	
		>1000-5000µg/day			488	SMD=-0.08 (-0.32, 0.16)	
		>5000µg/d			415	SMD=-0.43 (-1.24, 0.37)	
Valera-Gran, D. 2014 (Valencia) [48]	cohort study	400-1000µg/day	Periconception or mid-to-late pregnancy	Spain	542	SMD=-0.11 (-0.29, 0.08)	
		>1000-5000µg/day			517	SMD=-0.02 (-0.22, 0.18)	

		>5000μg/d			419	SMD=-0.20 (-0.59, 0.19)
Valera-Gran, D. 2017 (Asturias) [50]	cohort study	400-1000μg/day	Periconception or mid-to-late pregnancy	Norway	209	SMD=0.11 (-0.16, 0.39)
		>1000-5000μg/day			246	SMD=-0.16 (-0.41, 0.09)
Valera-Gran, D. 2017 (Guipuzkoa) [50]	cohort study	400-1000μg/day	Periconception or mid-to-late pregnancy	Norway	251	SMD=-0.36 (-0.71, -0.00)
		>1000-5000μg/day			326	SMD=-0.02 (-0.25, 0.21)
Valera-Gran, D. 2017 (Sabadell) [50]	case-control study	400-1000μg/day	Periconception or mid-to-late pregnancy	The United States	390	SMD=-0.06 (-0.36, 0.23)
		>1000-5000μg/day			406	SMD=-0.08 (-0.34, 0.18)
Valera-Gran, D. 2017 (Valencia) [50]	cohort study	400-1000μg/day	Periconception or mid-to-late pregnancy	Denmark	392	SMD=-0.05 (-0.27, 0.16)
		>1000-5000μg/day			396	SMD=0.19 (-0.03, 0.40)
Chatzi, L. 2012 [47]	Cohort Study	>1000~5000μg/d	Early pregnancy	Greece	419	SMD=0.06 (-0.24, 0.37)
		>5000μg/d			180	SMD=0.05 (-0.28, 0.39)

References

1. Wang, M.; Li, K.; Zhao, D.; Li, L. The association between maternal use of folic acid supplements during pregnancy and risk of autism spectrum disorders in children: a meta-analysis. *Molecular autism* **2017**, *8*, 51, doi:https://dx.doi.org/10.1186/s13229-017-0170-8.
2. Guo, B.-Q.; Li, H.-B.; Zhai, D.-S.; Ding, S.-B. Association of maternal prenatal folic acid intake with subsequent risk of autism spectrum disorder in children: A systematic review and meta-analysis. *Progress in neuro-psychopharmacology & biological psychiatry* **2019**, *94*, 109650, doi:https://dx.doi.org/10.1016/j.pnpbp.2019.109650.
3. Yu, X.-F.; Li, M.; Zheng, Y. [Association between maternal folate supplementation during pregnancy and the risk of autism spectrum disorder in the offspring: a Meta analysis]. *Zhongguo dang dai er ke za zhi = Chinese journal of contemporary pediatrics* **2017**, *19*, 286-291.
4. Liu, X.; Zou, M.; Sun, C.; Wu, L.; Chen, W.-X. Prenatal Folic Acid Supplements and Offspring's Autism Spectrum Disorder: A Meta-analysis and Meta-regression. *Journal of autism and developmental disorders* **2022**, *52*, 522-539, doi:https://dx.doi.org/10.1007/s10803-021-04951-8.
5. Jia, R.; Jin, S. Association between folic acid supplementation during pregnancy and the risk of autism spectrum disorder in the offspring: A meta-analysis. *Chinese Journal of Evidence-Based Medicine* **2021**, *21*, 1141-1147, http://dx.doi.org/10.7507/1672-2531.202105058.
6. Iglesias Vazquez, L.; Canals, J.; Arija, V. Review and meta-analysis found that prenatal folic acid was associated with a 58% reduction in autism but had no effect on mental and motor development. *Acta paediatrica (Oslo, Norway : 1992)* **2019**, *108*, 600-610, doi:https://dx.doi.org/10.1111/apa.14657.
7. Friel, C.; Leyland, A.H.; Anderson, J.J.; Havdahl, A.; Borge, T.; Shimonovich, M.; Dundas, R. Prenatal Vitamins and the Risk of Offspring Autism Spectrum Disorder: Systematic Review and Meta-Analysis. Nutrients 2021, 13, 2558 . https://doi.org/10.3390/nu13082558.
8. Li, M.; Francis, E.; Hinkle, S.N.; Ajjarapu, A.S.; Zhang, C. Preconception and Prenatal Nutrition and Neurodevelopmental Disorders: A Systematic Review and Meta-Analysis. Nutrients 2019, 11, 1628. https://doi.org/10.3390/nu11071628.
9. Chen, H.; Qin, L.; Gao, R.; Jin, X.; Cheng, K.; Zhang, S.; Hu, X.; Xu, W.; Wang, H. Neurodevelopmental effects of maternal folic acid supplementation: a systematic review and meta-analysis. *Critical reviews in food science and nutrition* **2023**, *63*, 3771-3787, doi:https://dx.doi.org/10.1080/10408398.2021.1993781.
10. Sampaio, A.C.; Matos, F.F.d.N.; Lopes, L.d.L.; Marques, I.M.M.; Tavares, R.M.; Fernandes, M.V.d.M.; Teixeira, M.R.V.d.S.; Brito, A.B.d.; Feitosa, A.C.; Guedes, T.O.; et al. Association of the Maternal Folic Acid Supplementation with the Autism Spectrum Disorder: A Systematic Review. *Associacao da suplementacao de acido folico materno com o transtorno do espectro do autismo: uma revisao sistematica.* **2021**, *43*, 775-781, doi:https://dx.doi.org/10.1055/s-0041-1736298.
11. Hoxha, B.; Hoxha, M.; Domi, E.; Gervasoni, J.; Persichilli, S.; Malaj, V.; Zappacosta, B. Folic Acid and Autism: A Systematic Review of the Current State of Knowledge. Cells 2021, 10, 1976. https://doi.org/10.3390/cells10081976.
12. Gao, Y.; Sheng, C.; Xie, R.-H.; Sun, W.; Asztalos, E.; Moddemann, D.; Zwaigenbaum, L.; Walker, M.; Wen, S.W. New Perspective on Impact of Folic Acid Supplementation during Pregnancy on Neurodevelopment/Autism in the Offspring Children - A Systematic Review. *PloS one* **2016**, *11*, e0165626, doi:https://dx.doi.org/10.1371/journal.pone.0165626.
13. DeVilbiss, E.A.; Gardner, R.M.; Newschaffer, C.J.; Lee, B.K. Maternal folate status as a risk factor for autism spectrum disorders: a review of existing evidence. *Br J Nutr* **2015**, *114*, 663-672, doi:10.1017/s0007114515002470.
14. Zhong, C.; Tessing, J.; Lee, B.K.; Lyall, K. Maternal Dietary Factors and the Risk of Autism Spectrum Disorders: A Systematic Review of Existing Evidence. *Autism research : official journal of the International Society for Autism Research* **2020**, *13*, 1634-1658, doi:https://dx.doi.org/10.1002/aur.2402.

15. Viswanathan, M.; Urrutia, R.P.; Hudson, K.N.; Middleton, J.C.; Kahwati, L.C. Folic Acid Supplementation to Prevent Neural Tube Defects: Updated Evidence Report and Systematic Review for the US Preventive Services Task Force. *JAMA* **2023**, *330*, 460-466, doi:https://dx.doi.org/10.1001/jama.2023.9864.
16. Vasconcelos, C.; Perry, I.S.; Gottfried, C.; Riesgo, R.; Castro, K. Folic acid and autism: updated evidences. *Nutritional Neuroscience* **2024**, *28*, 273-307, doi:10.1080/1028415x.2024.2367855.
17. Cheng, J.; Eskenazi, B.; Widjaja, F.; Cordero, J.F.; Hendren, R.L. Improving autism perinatal risk factors: A systematic review. *Medical hypotheses* **2019**, *127*, 26-33, doi:https://dx.doi.org/10.1016/j.mehy.2019.03.012.
18. Chmielewska, A.; Dziechciarz, P.; Gieruszczak-Białek, D.; Horvath, A.; Pieścik-Lech, M.; Ruszczyński, M.; Skórka, A.; Szajewska, H. Effects of prenatal and/or postnatal supplementation with iron, PUFA or folic acid on neurodevelopment: update. *Br J Nutr* **2019**, *122*, S10-s15, doi:10.1017/s0007114514004243.
19. Veena, S.R.; Gale, C.R.; Krishnaveni, G.V.; Kehoe, S.H.; Srinivasan, K.; Fall, C.H. Association between maternal nutritional status in pregnancy and offspring cognitive function during childhood and adolescence; a systematic review. *BMC Pregnancy Childbirth* **2016**, *16*, 220, doi:10.1186/s12884-016-1011-z.
20. Jalali Chimeh, F.; Aghaie, E.; Ghavi, S.; Fatahnia, R. Investigation of the Effects of Maternal Nutrition during Pregnancy on Cognitive Functions of Toddlers: A Systematic Review. *International journal of preventive medicine* **2024**, *15*, 15, doi:https://dx.doi.org/10.4103/ijpvm.ijpvm_124_22.
21. Freedman, R.; Hunter, S.K.; Hoffman, M.C. Prenatal Primary Prevention of Mental Illness by Micronutrient Supplements in Pregnancy. *The American journal of psychiatry* **2018**, *175*, 607-619, doi:https://dx.doi.org/10.1176/appi.ajp.2018.17070836.
22. Chamova, R.; Toneva, A.; Brajkova, R.; Pancheva, R. Impact of Maternal Nutrition on Child Neurodevelopment: Insights from Recent Studies. Biomed. Rev. 2023, 34, 109–119.https://doi.org/10.14748/bmr.v34.9619.
23. Chen, Q.; Huang, L.-X.; Xu, W.-J.; Chen, H.; Zhong, J.-Q.; Zeng, C.-X. Research on the prevalence and the risk factors of autism spectrum disorder from 1.5 to 3 years old in Zhuhai city. Chin. J. Child Health Care 2014, 22, 649-651, doi:Cnki:Sun:Erto.0.2014-06-030. (In Chinese)
24. Li, X.; Ling, Z.-Y.; Wang, J.-M.; Yang, S.-P.; Qin, Y.-Y.; Xie, S.-N.; Yang, S.-B.; Zhang, J.Periconceptional Risk Factors for Childhood Autism:A 1 ∶ 1 Matched Case-control Study. Acta Med. Univ. Sci. Technol. Huazhong 2015, 44, 357-361, doi:Cnki:Sun:Tjyx.0.2015-03-026. (In Chinese)
25. Su, Y.-Y.. Effect of Environmental Risk Factor on Children with Autistic disorder and Mental Retardation. Master's degree, Tianjin Medical University, 2012. doi:10.7666/d.Y2232222. (In Chinese)
26. Jiang, H.; Liu, L.; Sun, D.L.; Yin, X.N.; Chen, Z.D.; Wu, C.A.; Chen, W.Q. [Interaction between passive smoking and folic acid supplement during pregnancy on autism spectrum disorder behaviors in children aged 3 years]. *Zhonghua Liu Xing Bing Xue Za Zhi* **2016**, *37*, 940-944, doi:10.3760/cma.j.issn.0254-6450.2016.07.007.
27. Nilsen, R.M.; Surén, P.; Gunnes, N.; Alsaker, E.R.; Bresnahan, M.; Hirtz, D.; Hornig, M.; Lie, K.K.; Lipkin, W.I.; Reichborn-Kjennerud, T.; et al. Analysis of self-selection bias in a population-based cohort study of autism spectrum disorders. *Paediatr Perinat Epidemiol* **2013**, *27*, 553-563, doi:10.1111/ppe.12077.
28. Surén, P.; Roth, C.; Bresnahan, M.; Haugen, M.; Hornig, M.; Hirtz, D.; Lie, K.K.; Lipkin, W.I.; Magnus, P.; Reichborn-Kjennerud, T.; et al. Association between maternal use of folic acid supplements and risk of autism spectrum disorders in children. *Jama* **2013**, *309*, 570-577, doi:10.1001/jama.2012.155925.
29. Schmidt, R.J.; Tancredi, D.J.; Ozonoff, S.; Hansen, R.L.; Hartiala, J.; Allayee, H.; Schmidt, L.C.; Tassone, F.; Hertz-Picciotto, I. Maternal periconceptional folic acid intake and risk of autism spectrum disorders and developmental delay in the CHARGE (CHildhood Autism Risks from Genetics and Environment) case-control study. *Am J Clin Nutr* **2012**, *96*, 80-89, doi:10.3945/ajcn.110.004416.
30. Virk, J.; Liew, Z.; Olsen, J.; Nohr, E.A.; Catov, J.M.; Ritz, B. Preconceptional and prenatal supplementary folic acid and multivitamin intake and autism spectrum disorders. *Autism* **2016**, *20*, 710-718, doi:10.1177/1362361315604076.

31. Sun, Y.; Shao, T.; Yao, Y.; Tao, H.; Ni, L.; Yan, S.; Gu, C.; Cao, H.; Huang, K.; Tao, F. [Pregnancy-related anxiety and subthreshold autism trait in preschool children based a birth cohort study]. *Zhonghua Yu Fang Yi Xue Za Zhi* **2016**, *50*, 118-122, doi:10.3760/cma.j.issn.0253-9624.2016.02.004.
32. Desoto, M.; Robert, H. Synthetic folic acid supplementation during pregnancy may increase the risk of developing autism. *Journal of Pediatric Biochemistry* **2016**, *02*, 251-261.
33. DeVilbiss, E.A.; Magnusson, C.; Gardner, R.M.; Rai, D.; Newschaffer, C.J.; Lyall, K.; Dalman, C.; Lee, B.K. Antenatal nutritional supplementation and autism spectrum disorders in the Stockholm youth cohort: population based cohort study. *Bmj* **2017**, *359*, j4273, doi:10.1136/bmj.j4273.
34. Levine, S.Z.; Kodesh, A.; Viktorin, A.; Smith, L.; Uher, R.; Reichenberg, A.; Sandin, S. Association of Maternal Use of Folic Acid and Multivitamin Supplements in the Periods Before and During Pregnancy With the Risk of Autism Spectrum Disorder in Offspring. *JAMA Psychiatry* **2018**, *75*, 176-184, doi:10.1001/jamapsychiatry.2017.4050.
35. Strøm, M.; Granström, C.; Lyall, K.; Ascherio, A.; Olsen, S.F. Research Letter: Folic acid supplementation and intake of folate in pregnancy in relation to offspring risk of autism spectrum disorder. *Psychol Med* **2018**, *48*, 1048-1054, doi:10.1017/s0033291717002410.
36. Schmidt, R.J.; Iosif, A.M.; Guerrero Angel, E.; Ozonoff, S. Association of Maternal Prenatal Vitamin Use With Risk for Autism Spectrum Disorder Recurrence in Young Siblings. *JAMA Psychiatry* **2019**, *76*, 391-398, doi:10.1001/jamapsychiatry.2018.3901.
37. Schmidt, R.J.; Kogan, V.; Shelton, J.F.; Delwiche, L.; Hansen, R.L.; Ozonoff, S.; Ma, C.C.; McCanlies, E.C.; Bennett, D.H.; Hertz-Picciotto, I.; et al. Combined Prenatal Pesticide Exposure and Folic Acid Intake in Relation to Autism Spectrum Disorder. *Environ Health Perspect* **2017**, *125*, 097007, doi:10.1289/ehp604.
38. Li, Y.M.; Shen, Y.D.; Li, Y.J.; Xun, G.L.; Liu, H.; Wu, R.R.; Xia, K.; Zhao, J.P.; Ou, J.J. Maternal dietary patterns, supplements intake and autism spectrum disorders: A preliminary case-control study. *Medicine (Baltimore)* **2018**, *97*, e13902, doi:10.1097/md.0000000000013902.
39. Tan, M.; Yang, T.; Zhu, J.; Li, Q.; Lai, X.; Li, Y.; Tang, T.; Chen, J.; Li, T. Maternal folic acid and micronutrient supplementation is associated with vitamin levels and symptoms in children with autism spectrum disorders. *Reprod Toxicol* **2020**, *91*, 109-115, doi:10.1016/j.reprotox.2019.11.009.
40. Sharman Moser, S.; Davidovitch, M.; Rotem, R.S.; Chodick, G.; Shalev, V.; Koren, G. High dose folic acid during pregnancy and the risk of autism; The birth order bias: A nested case-control study. *Reprod Toxicol* **2019**, *89*, 173-177, doi:10.1016/j.reprotox.2019.07.083.
41. Braun, J.M.; Froehlich, T.; Kalkbrenner, A.; Pfeiffer, C.M.; Fazili, Z.; Yolton, K.; Lanphear, B.P. Brief report: are autistic-behaviors in children related to prenatal vitamin use and maternal whole blood folate concentrations? *J Autism Dev Disord* **2014**, *44*, 2602-2607, doi:10.1007/s10803-014-2114-x.
42. Goodrich, A.J.; Volk, H.E.; Tancredi, D.J.; McConnell, R.; Lurmann, F.W.; Hansen, R.L.; Schmidt, R.J. Joint effects of prenatal air pollutant exposure and maternal folic acid supplementation on risk of autism spectrum disorder. *Autism Res* **2018**, *11*, 69-80, doi:10.1002/aur.1885.
43. Schmidt, R.J.; Hansen, R.L.; Hartiala, J.; Allayee, H.; Schmidt, L.C.; Tancredi, D.J.; Tassone, F.; Hertz-Picciotto, I. Prenatal vitamins, one-carbon metabolism gene variants, and risk for autism. *Epidemiology* **2011**, *22*, 476-485, doi:10.1097/EDE.0b013e31821d0e30.
44. Al-maqati, T.N.; Al-Otaibi, N.M.; Al-Merbati, L.S.; Al-Dossary, D.M. Prenatal factors influencing the risk of autism spectrum disorder. *Advances in Neurodevelopmental Disorders* **2021**, *5*, 71-76.
45. Julvez, J.; Fortuny, J.; Mendez, M.; Torrent, M.; Ribas-Fitó, N.; Sunyer, J. Maternal use of folic acid supplements during pregnancy and four-year-old neurodevelopment in a population-based birth cohort. *Paediatr Perinat Epidemiol* **2009**, *23*, 199-206, doi:10.1111/j.1365-3016.2009.01032.x.

46. del Río Garcia, C.; Torres-Sánchez, L.; Chen, J.; Schnaas, L.; Hernández, C.; Osorio, E.; Portillo, M.G.; López-Carrillo, L. Maternal MTHFR 677C>T genotype and dietary intake of folate and vitamin B(12): their impact on child neurodevelopment. *Nutr Neurosci* **2009**, *12*, 13-20, doi:10.1179/147683009x388913.
47. Chatzi, L.; Papadopoulou, E.; Koutra, K.; Roumeliotaki, T.; Georgiou, V.; Stratakis, N.; Lebentakou, V.; Karachaliou, M.; Vassilaki, M.; Kogevinas, M. Effect of high doses of folic acid supplementation in early pregnancy on child neurodevelopment at 18 months of age: the mother-child cohort 'Rhea' study in Crete, Greece. *Public Health Nutr* **2012**, *15*, 1728-1736, doi:10.1017/s1368980012000067.
48. Valera-Gran, D.; García de la Hera, M.; Navarrete-Muñoz, E.M.; Fernandez-Somoano, A.; Tardón, A.; Julvez, J.; Forns, J.; Lertxundi, N.; Ibarluzea, J.M.; Murcia, M.; et al. Folic acid supplements during pregnancy and child psychomotor development after the first year of life. *JAMA Pediatr* **2014**, *168*, e142611, doi:10.1001/jamapediatrics.2014.2611.
49. Polańska, K.; Muszyński, P.; Sobala, W.; Dziewirska, E.; Merecz-Kot, D.; Hanke, W. Maternal lifestyle during pregnancy and child psychomotor development - Polish Mother and Child Cohort study. *Early Hum Dev* **2015**, *91*, 317-325, doi:10.1016/j.earlhumdev.2015.03.002.
50. Valera-Gran, D.; Navarrete-Muñoz, E.M.; Garcia de la Hera, M.; Fernández-Somoano, A.; Tardón, A.; Ibarluzea, J.; Balluerka, N.; Murcia, M.; González-Safont, L.; Romaguera, D.; et al. Effect of maternal high dosages of folic acid supplements on neurocognitive development in children at 4-5 y of age: the prospective birth cohort Infancia y Medio Ambiente (INMA) study. *Am J Clin Nutr* **2017**, *106*, 878-887, doi:10.3945/ajcn.117.152769.
51. Campoy, C.; Escolano-Margarit, M.V.; Ramos, R.; Parrilla-Roure, M.; Csábi, G.; Beyer, J.; Ramirez-Tortosa, M.C.; Molloy, A.M.; Decsi, T.; Koletzko, B.V. Effects of prenatal fish-oil and 5-methyltetrahydrofolate supplementation on cognitive development of children at 6.5 y of age. *Am J Clin Nutr* **2011**, *94*, 1880s-1888s, doi:10.3945/ajcn.110.001107.
52. Czeizel, A.E.; Dobó, M. Postnatal somatic and mental development after periconceptional multivitamin supplementation. *Arch Dis Child* **1994**, *70*, 229-233, doi:10.1136/adc.70.3.229.
53. Dobó, M.; Czeizel, A.E. Long-term somatic and mental development of children after periconceptional multivitamin supplementation. *Eur J Pediatr* **1998**, *157*, 719-723, doi:10.1007/s004310050922.
